# A unique self-truncation of bacterial GH5 endoglucanases leads to enhanced activity and thermostability

**DOI:** 10.1186/s12915-022-01334-y

**Published:** 2022-06-09

**Authors:** Mei-Huey Wu, Mu-Rong Kao, Chen-Wei Li, Su-May Yu, Tuan-Hua David Ho

**Affiliations:** 1 Department of Biotechnology and Bioindustry Sciences, National Cheng Kung University, Tainan, 701 Taiwan, Republic of China; 2 Institute of Plant and Microbial Biology, Academia Sinica, Nankang, Taipei, 115 Taiwan, Republic of China; 3 Institute of Molecular Biology, Academia Sinica, Nankang, Taipei, 115 Taiwan, Republic of China; 4 Biotechnology Research Center, National Chung Hsing University, Taichung, 402 Taiwan, Republic of China; 5 Department of Life Sciences, National Chung Hsing University, Taichung, 402 Taiwan, Republic of China

**Keywords:** Endoglucanase, Self-truncation, GH family 5, *Geobacillus*

## Abstract

**Background:**

β-1,4-endoglucanase (EG) is one of the three types of cellulases used in cellulose saccharification during lignocellulosic biofuel/biomaterial production. GsCelA is an EG secreted by the thermophilic bacterium *Geobacillus* sp. 70PC53 isolated from rice straw compost in southern Taiwan. This enzyme belongs to glycoside hydrolase family 5 (GH5) with a TIM-barrel structure common among all members of this family. GsCelA exhibits excellent lignocellulolytic activity and thermostability. In the course of investigating the regulation of this enzyme, it was fortuitously discovered that GsCelA undergoes a novel self-truncation/activation process that appears to be common among GH5 enzymes.

**Results:**

Three diverse Gram-positive bacterial GH5 EGs, but not a GH12 EG, undergo an unexpected self-truncation process by removing a part of their C-terminal region. This unique process has been studied in detail with GsCelA. The purified recombinant GsCelA was capable of removing a 53-amino-acid peptide from the C-terminus. Natural or engineered GsCelA truncated variants, with up to 60-amino-acid deletion from the C-terminus, exhibited higher specific activity and thermostability than the full-length enzyme. Interestingly, the C-terminal part that is removed in this self-truncation process is capable of binding to cellulosic substrates of EGs. The protein truncation, which is pH and temperature dependent, occurred between amino acids 315 and 316, but removal of these two amino acids did not stop the process. Furthermore, mutations of E142A and E231A, which are essential for EG activity, did not affect the protein self-truncation process. Conversely, two single amino acid substitution mutations affected the self-truncation activity without much impact on EG activities. In *Geobacillus* sp. 70PC53, the full-length GsCelA was first synthesized in the cell but progressively transformed into the truncated form and eventually secreted. The GsCelA self-truncation was not affected by standard protease inhibitors, but could be suppressed by EDTA and EGTA and enhanced by certain divalent ions, such as Ca^2+^, Mg^2+^, and Cu^2+^.

**Conclusions:**

This study reveals novel insights into the strategy of Gram-positive bacteria for directing their GH5 EGs to the substrate, and then releasing the catalytic part for enhanced activity via a spontaneous self-truncation process.

**Supplementary Information:**

The online version contains supplementary material available at 10.1186/s12915-022-01334-y.

## Background

Lignocellulosic biomass is the most abundant biological material on earth and also an important sustainable source for biofuels and biomaterials available to humans [1]. Lignocellulosic biomass has a complex structure, with crystalline and amorphous cellulose, hemicellulose, and lignin as major components. Cellulose and hemicellulose are polysaccharides of energy-rich sugars that can be converted into renewable energy and commodity chemicals [2]. At least 6 × 10^12^ tons of agricultural residues are generated globally each year [3]. A large portion of the resources are from annual crop residues such as rice straw, wheat straw, corn stover, and sugarcane bagasse [4].

Several steps, including pretreatment, hydrolysis, and fermentation, are involved in converting agricultural waste feedstock into cellulosic ethanol or other readily usable forms of biofuels. The most challenging step in this process is the effective conversion of pretreated cellulosic materials to fermentable sugars. Specifically, this process requires three classes of enzymes: (1) endoglucanases (EG, EC 3.2.1.4), which randomly cleave internal bonds in amorphous regions of cellulose to yield cello-oligosaccharides and small amounts of glucose; (2) exoglucanases (EXO, EC 3.2.1.91), which exolytically attack the reducing or non-reducing end of celluloses to yield cellobiose; and (3) β-glucosidases (BGL, EC 3.2.1.21), which hydrolyze cellobiose to form glucose. Although these enzymes are currently available commercially, their prices are high, which contributes to a significant part of the overall production cost of cellulosic biofuels.

Carbohydrate-active enzymes can be classified according to their protein sequence and structure. Glycoside hydrolase family 5 (GH5) is one of the largest families among all GHs, and it acts on a variety of glycosidic bonds [5]. GH5 enzymes have broad substrate specificity and are widely distributed in all species. About 80% of all known GH5 sequences are classified into 53 subfamilies; they are listed in the CAZy (Carbohydrate-Active enZYmes) database (http://cazy.org). The 3-D structure of GH5 enzymes was determined as a classical (α/β)_8_ TIM-barrel [6, 7]. The two catalytic residues in GH5 enzymes are both glutamic acids, one as a nucleophile and the other as the catalytic proton donor.

Some cellulases, including GH5 EGs, have two major structural domains: one for catalytic activity and the other for substrate binding. The carbohydrate-binding module (CBM) assists cellulase binding toward insoluble substrates, resulting in increased hydrolysis efficiency [8]. Engineered deletion of CBM in recombinant EGs improves the thermostability and enzyme activity toward amorphous cellulose [9, 10]. For example, an earlier study showed the molecular weight of a native secreted *Bacillus* EG decreased upon prolonged culture of the bacterium, and this phenomenon was due to a protease-dependent cleavage at the C-terminal region that may contain a CBM [11, 12]. Therefore, the recombinant *Bacillus* EG recovered from *Escherichia coli* (*E. coli*) is a truncated form due to its cleavage in the periplasmic space by an *E. coli* protease [13].

Protein self-cleavage is a post-translational modification well described in proteases such as trypsin and also in other proteins carrying a proteolytic domain. For example, the signaling protein SpoIVB plays an important role in regulating spore formation in *Bacillus subtilis*. This protein contains serine peptidase activity involved in the self-cleavage, and the cleaved and mature form of SpoIVB can later activate a zinc metalloprotease [14]. Human chloride channel calcium-activated 1 (CLCA1) has a metalloprotease domain at its N-terminus and a conserved cleavage site at its C-terminus. The removal of the C-terminal region by self-cleavage is indispensable for activating the chloride channel [15]. NopE1, a secreted protein from the Gram-negative symbiont bacterium *Bradyrhizobium japonicum*, also undergoes self-cleavage process by a specific calcium-binding EF-hand-like motif in its C-terminal region [16].

In our previous studies, we isolated a thermophilic bacterium *Geobacillus* sp. 70PC53 secreting a highly active and thermostable EG, GsCelA, from rice straw compost supplemented with pig manure [17]. GsCelA shares the same structural characteristics as other GH5 EGs by its highly conserved catalytic glutamic acid residues at the active site and a (α/β)_8_ TIM-barrel structure without CBM [18].

In this study, we fortuitously observed that a smaller form of GsCelA appeared after long-term storage of highly purified enzyme. Furthermore, the smaller form had lost the C-terminal region yet still retained the catalytic domain and showed higher enzyme activity and thermostability than the full-length enzyme. We further explored the conditions that regulate this intriguing self-truncation process and discovered that it appears to be novel and independent of a separate protease. Furthermore, this phenomenon is not unique to GsCelA, as at least two other GH5 EGs in two different subfamilies also undergo similar self-truncation.

## Results

### Recombinant GsCelA self-truncates spontaneously

**Fig. 1:**
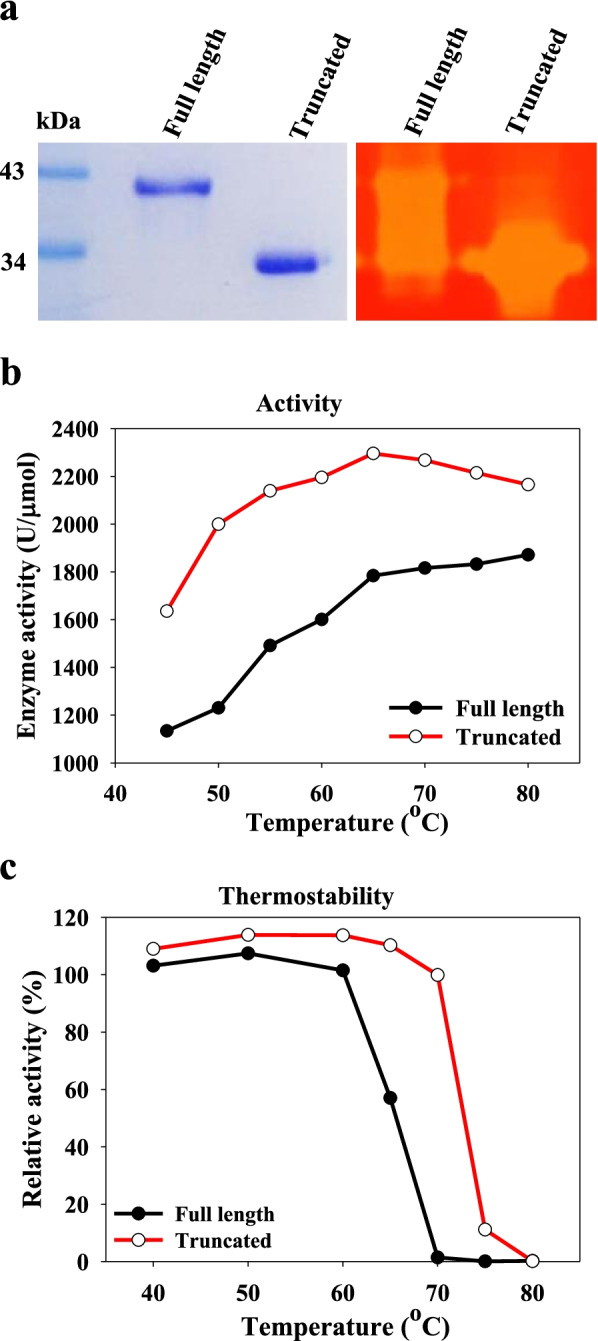
GsCelA becomes more active after spontaneous truncation. **a** Purified GsCelA becomes truncated form after a long-term storage in 50 mM sodium phosphate, pH 7 at 4 °C (in left). CMC zymography of truncated and full-length GsCelA (in right). **b** Test tube assays of enzyme activity using CMC as substrate at different temperatures in 50 mM sodium acetate, pH 5 at 65 °C. **c** Thermostability assays with full-length and truncated forms GsCelA pre-incubated 6 h at different temperatures (45 to 80 °C. One enzyme unit (U) is defined as 1 μmol reducing sugar, i.e., glucose equivalent, produced per minute

GsCelA is a *Geobacillus* GH5 subfamily 2 (GH5-2) EG that shares only 63% sequence identity with its nearest homologs. Thus, phylogenetic study also revealed that GsCelA is in a unique clade distant from other *Geobacillus* and *Bacillus* enzymes (Supplementary Figure 1, Additional file 1; Supplementary Table 1, Additional file 1). The fresh prepared highly purified full-length GsCelA (FL-GsCelA) had a molecular weight of 40 kDa on SDS-PAGE (Fig. 1a, left panel). After long-term incubation (about 4 weeks) of purified FL-GsCelA at 4 °C, a smaller protein appeared at 35 kDa. When the CMC (carboxymethyl cellulose) zymography was performed to compare the enzymatic activity of these two samples collected before and after the long-term incubation, the sample containing the smaller protein showed a larger clear zone on the gel, which indicates higher EG activity even with lower protein content than for FL-GsCelA (Fig. 1a, right panel). To exclude the potential artifactual effect of cloning, such as unexpected mutations generated by PCR amplification, we used GsCelA-specific primers to amplify GsCelA gene from the native bacterium, *Geobacillus* sp. 70PC53. Results of DNA sequence alignment confirmed that the coding sequence for FL-GsCelA, which is used for heterologous expression of this enzyme in *E. coli*, is identical to the sequence in the genome of *Geobacillus* sp. 70PC53 (Supplementary Figure 2, Additional file 1).

We compared both enzyme activity and thermostability between FL-GsCelA and the smaller 35-kDa protein. The smaller 35-kDa protein exhibited higher EG activity than the FL-GsCelA at all temperatures tested with 1% CMC used as the substrate (Fig. 1b). After 6 h of incubation at 60–70 °C, the 35-kDa form remained more thermostable than FL-GsCelA, with only a slight decrease of relative activity (Fig. 1c). The N-terminal sequence of the 35-kDa protein, MERTPVEENG, as determined by the Edman degradation method, was the same as for FL-GsCelA (Supplementary Figure 3a, Additional file 1). LC-MS-MS was used to analyze the smaller 35-kDa protein, and peptide fragments obtained after digestion by trypsin or chymotrypsin were identified. These peptide sequences covered the major part of FL-GsCelA except the C-terminal region from 316 to 368 amino acids (Supplementary Figure 3a, Additional file 1), which suggests that the small protein is derived from GsCelA and appears to be the C-terminal region truncation product. High-resolution mass spectra obtained from whole molecular weight analysis showed a unique single peak for both FL-GsCelA and truncated GsCelA (Supplementary Figure 3b and 3c, Additional file 1). The molecular weight of the highly purified protein was 42,140 Da for FL-GsCelA with a C-terminal 6-His tag and 35,491 Da for the smaller form GsCelA, which coincides with the molecular weight of the first 315-amino-acid protein from the N-terminus (Supplementary Figure 3c, Additional file 1). From these results, we reasoned that there must be a truncation of GsCelA appearing between amino acid residues K315 and G316.

### Other GH5 endoglucanases display similar self-truncation phenomenon

**Fig. 2:**
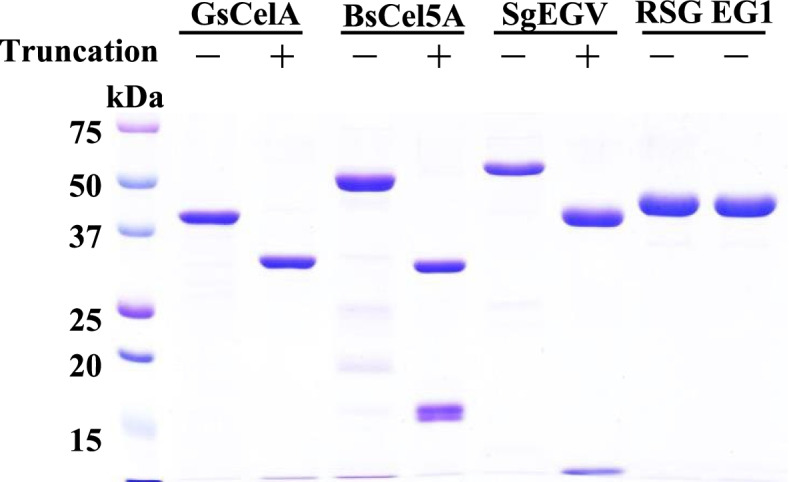
GH5 endoglucanase secreted from Gram-positive bacteria show similar self-truncation process. GH5 endoglucanases *Geobacillus* sp. 70PC53 GsCelA, *Streptococcus* sp. SgEGV and *Bacillus subtilis* strain 168 BsCel5A and *Micromonospora* (or *Thermobispora*) GH12 endoglucanase RSC EG1 were incubated in 50 mM sodium acetate, pH 5 at 4 °C for 120 h to assay their self-truncation ability. Four micrograms of purified enzymes were applied to each lane of the gel

To evaluate whether the aforementioned truncation phenomenon is common among GH5 EGs, we expressed two other GH5 EGs and one GH12 EG in *E. coli* and purified them by the same experimental procedure used for FL-GsCelA. BsCel5A is a GH5 subfamily 2 (GH5-2) EG with a CBM family 3 (CBM3) sequence in the C-terminal region and is secreted from *Bacillus subtilis* strain 168 [19]. The second GH5 EG, SgEGV, is a GH5 subfamily 4 (GH5-4) EG secreted from *Streptococcus* sp. without a known CBM (Supplementary Figure 1, Additional file 1). RSC EG1 is a GH12 EG discovered by metagenomics analysis of rice straw compost microbial community, likely secreted from *Micromonospora* or *Thermobispora* [20]. FL-GsCelA has a sequence similarity of 37.9% with BsCel5A, 13.4% with SgEGV, and 14% with RSC EG1. The self-truncation experiment was performed with fresh highly purified enzymes at 25 °C for 72 h. SDS-PAGE revealed protein truncation for all three GH5 EGs (i.e., GsCelA, BsCel5A and SgEGV), but GH12 RSC EG1 remained unchanged after 120-h incubation at 25 °C (Fig. 2). BsCel5A (52.7 kDa) and SgEGV (57 kDa) were truncated into two fragments each. BsCel5A was truncated into 33.8- and 17.5-kDa fragments, and SgEGV was cut into 42- and 15-kDa fragments (Fig. 2). MS analyses revealed that the cleavage site for BsCel5A was between T305 and K306 (Supplementary Figure 4a, Additional file 1), and the cleavage site for SgEGV was likely between N369 and T370 (Supplementary Figure 4b, Additional file 1). Therefore, the spontaneous truncation process was observed in at least three different GH5 EGs in GH5-2 and GH5-4 subfamilies but not for a GH12 EG, and this process is likely to be associated with GH5 EGs containing a common TIM-barrel structure.

### Enzyme activity and thermostability are enhanced by C-terminal truncation in GsCelA

**Fig. 3:**
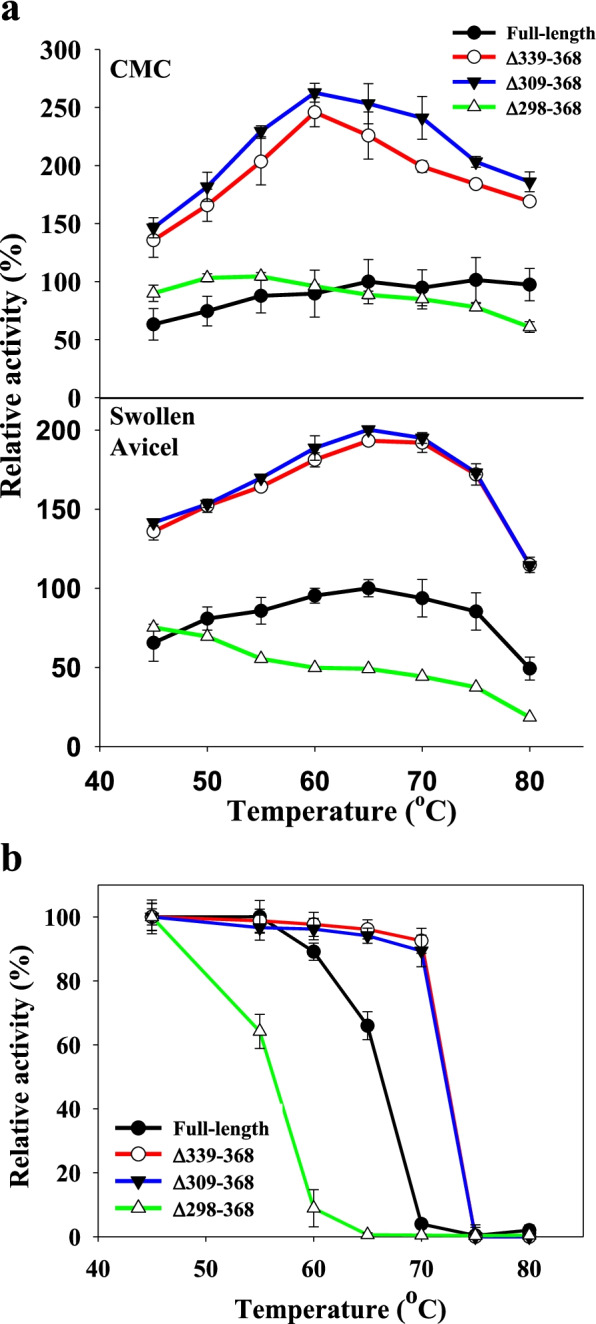
Engineered truncation of up to 60 amino acid residues from C-terminus improved GsCelA activity and thermostability. **a** Enzyme activities at different temperatures with CMC or swollen Avicel used as substrate in 50 mM sodium acetate, pH 5. **b** Thermostability assays with samples pre-incubated at different temperatures. Data are mean ± SD. One enzyme unit (U) is defined as 1 μmole reducing sugar, i.e., equivalent to glucose, produced per minute. All experiments were performed in triplicate

**Fig. 4:**
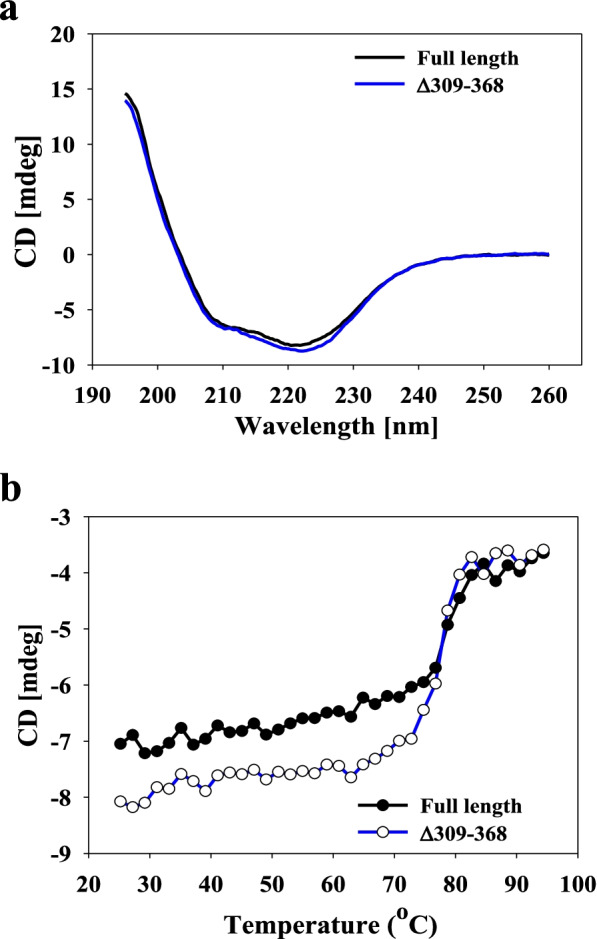
Comparison of circular dichroism (CD) profiles between FL-GsCelA and the C-terminal ∆309-368 mutant. CD analysis of **a** secondary structure and **b** melting temperature

To test the effect of C-terminal truncation on the properties of GsCelA, we genetically engineered three GsCelA mutants with different degrees of C-terminal truncation (Δ339–368, Δ309–368, and Δ298–368). Enzyme activities of these artificially truncated GsCelA mutants were determined by using CMC as a soluble substrate and swollen Avicel as an insoluble substrate. Deletion of up to 60 amino acids from the C-terminus (i.e., Δ339–368 and Δ309–368) increased enzyme activity by up to 210% at 65 °C (Fig. 3a) and improved enzyme thermostability (Fig. 3b). However, deletion of more than 60 amino acids (i.e., Δ298–368) decreased both enzyme activity and thermostability (Fig. 3). The Δ298–368 mutant showed similar enzyme activity as FL-GsCelA below 60 °C but lower enzyme activity at > 65 °C (Fig. 3a). Using swollen Avicel as a substrate, the Δ339–368 and Δ309–368 mutants showed 2-fold higher activity than FL-GsCelA in a broad range of temperatures from 45 to 80 °C, whereas the Δ298–368 mutant did not maintain the same level of enzyme activity with increasing temperature (Fig. 3a). In the thermostability test, FL-GsCelA retained 65% of enzyme activity after incubation at 65 °C for 6 h. However, the Δ339–368 and Δ309–368 mutants were able to retain 95% enzyme activity after incubation at 70 °C for 6 h. On the other hand, the Δ298–368 mutant showed greatly decreased enzyme activity at > 60 °C (Fig. 3b). C-terminal truncations did not cause major changes of protein structure because FL-GsCelA and the Δ309–368 mutant had similar secondary structure (Fig. 4a) and melting temperature (Fig. 4b) as determined by circular dichroism (CD) analyses. The parameter T_50_ reflects the enzyme’s tolerance to heat stress, whereas TA_50_ reveals the enzyme’s ability to function at elevated temperature [21]. The T_50_ values for FL-GsCelA and the Δ309–368 mutant were 74 °C and 77 °C, respectively (Supplementary Figure 5a, Additional file 1). However, the Δ309–368 mutant had a TA_50_ above 100 °C, which was significantly higher than the 86 °C for FL-GsCelA (Supplementary Figure 5b, Additional file 1).

### Effect of C-terminal deletions on GsCelA self-truncation

**Fig. 5:**
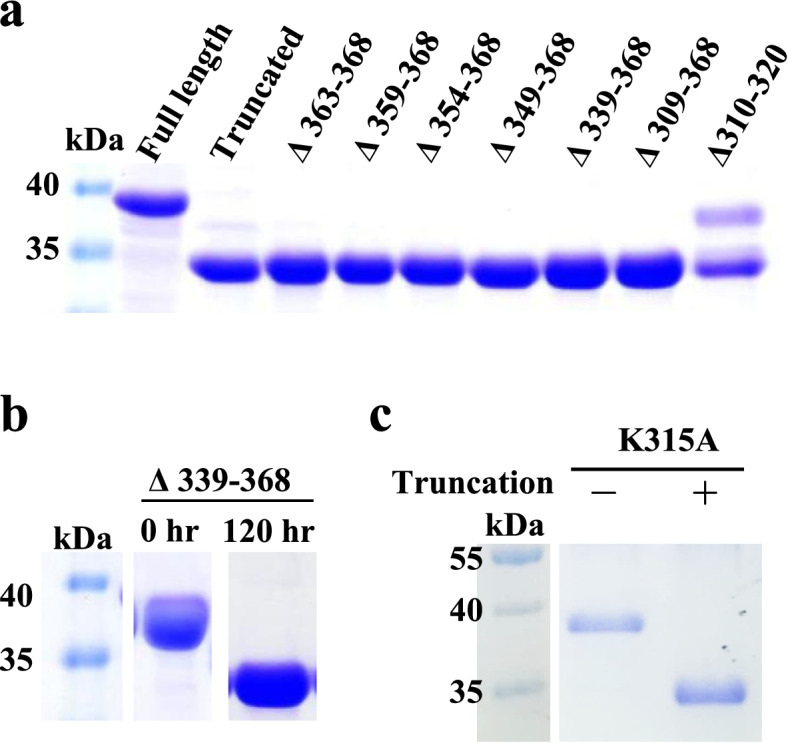
GsCelA self-truncation still takes place with different C-terminal mutants. **a** The truncation process was assayed with GsCelA mutants carrying partial deletion at C-terminus after incubation at 25 °C for 120 h. **b** Δ339-368 mutant with 30 amino acids near the C-terminus removed was still capable of truncation. **c** Single mutation of K315A, the truncation site, did not affect the truncation process after incubation at 25 °C for 120 h. Labels of “+” and “−” indicate samples with or without self-truncation, respectively. Full-length and truncated GsCelA, which correspond to samples before and after incubation, were used as positive and negative controls, respectively. Marker and protein profiles in b and c were cut from the same SDS-PAGE gel

Because GsCelA self-truncation takes place at its C-terminal region, we constructed four additional C-terminal truncation mutants of GsCelA (Δ363–368, Δ359–368, Δ354–368, Δ349–368) and one internal deletion mutant Δ310–320 covering the truncation site at K315/G316. Truncation tests were performed at 25 °C with all of these five new mutants together with the three C-terminal mutants described in the previous section. Samples were collected from these mutants every 24 h followed by SDS-PAGE analysis. As expected, Δ309–368 mutant did not undergo self-truncation because the deletion in this mutant has totally removed the truncation site at K315/G316 and its flanking regions (Fig 5a). Interestingly, Δ339–368 was still capable of truncation even without the last 30 amino acids from the C-terminus (Fig. 5b). Similarly, none of the new C-terminal shorter deletions was able to suppress the GsCelA truncation process. However, the internal deletion Δ310–320, without the cleavage site K315/G316 and its flanking region, was able to slow down the process, with incomplete truncation even after 120 h (Fig. 5a, c). K315 was chosen for site-directed mutagenesis because it is right at the truncation site. However, the self-truncation was still observed when K315 was substituted by alanine (A), which indicates that the truncation process does not depend on this amino acid residue (Fig. 5c). Examined by LC-MS analysis, the mutant Δ310–320 has different cutting sites, resulting in different lengths of truncated protein ranging from 312 to 319 amino acids (Supplementary Figure 6, Additional file 1). This result suggests that the slower truncation is also less precise than the one observed in FL-GsCelA. Thus, GsCelA truncation did not recognize the specific amino acid sequence near the truncation site, yet always generated truncated proteins with similar size.

### GsCelA self-truncation process is independent of EG activity but inhibited by substrate and an end product of EG

**Fig. 6:**
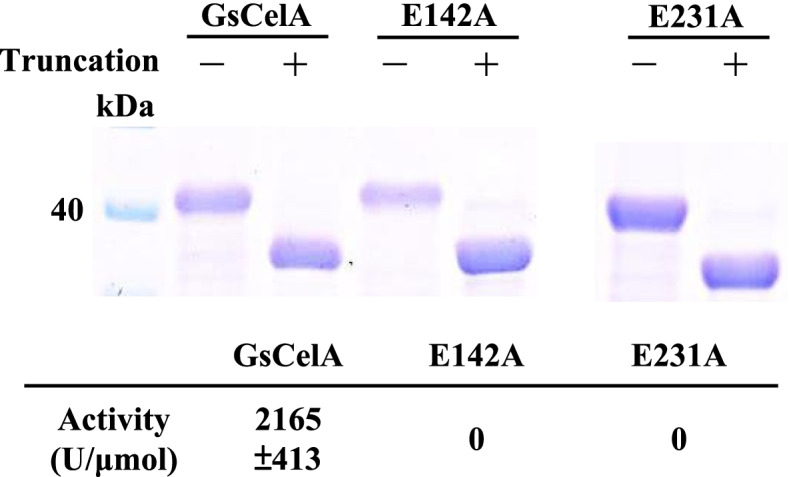
GsCelA self-truncation process is not dependent on endoglucanase activity. Self-truncation assays were carried with wild-type and mutants GsCelA at 25 a for 120 h. Enzyme activity was measured by CMC assay in 50 mM sodium acetate, pH 5 at 65 °C

From structure/function analyses of GsCelA and other GH5 EGs, E142 and E231 have been identified as catalytic amino acid residues essential for the EG glycolytic reaction [18]. Site-directed mutagenesis was used to substitute these two specific E142 and E231 with alanine (A) to suppress the EG activity of GsCelA. As expected, both E142A and E231A mutants did not exhibit any EG activity (Fig. 6). However, the self-truncation process still took place for these mutants under the same experimental conditions as for the wild-type enzyme, and the truncated proteins from wild-type GsCelA and mutants E142A and E231A appeared to have the same size (Fig. 6). Surprisingly, the self-truncation process was inhibited by 1% and 2% CMC, or 2% and 4% cellobiose (Supplementary Figure 7, Additional file 1). These results suggest that although the self-truncation process does not depend on the EG catalytic activity, it can still be inhibited by its substrate and product.

### GsCelA truncation process is affected by pH and temperature

**Fig. 7:**
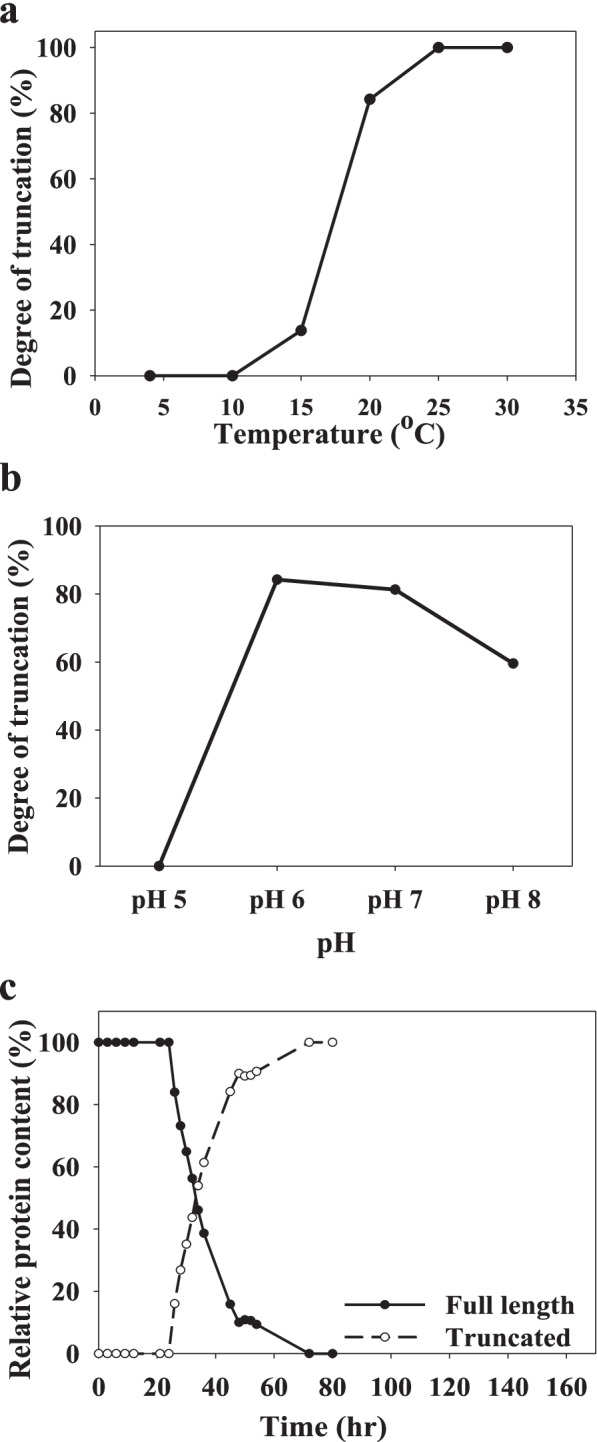
GsCelA self-truncation is temperature- and pH-dependent. **a** GsCelA self-truncation at different temperatures and **b** at different pH for 48 h incubation. **c** Time course of self-truncation process at 25 °C and pH 7. Error bars represented standard deviation values

We first observed GsCelA self-truncation after long-term storage (usually for weeks) of purified enzyme in 20 mM sodium phosphate (pH 7) at 4 °C. To determine whether this truncation process is pH- and temperature-dependent, we incubated FL-GsCelA from 4 to 30 °C and at pH 5 to 8. After a 48-h incubation, self-truncation was not observed at temperatures lower than 10 °C but became progressively more truncated at higher temperatures until reaching 100% at 25 °C (Fig. 7a). The self-truncation also appeared at pH between 5 and 8 (Fig. 7b). We monitored the progress of GsCelA self-truncation at 25 °C, pH 7 by collecting samples at short time intervals (Fig. 7c). This truncation process reached 50% in ~30 h and was completed after 70 h.

### GsCelA self-truncation is independent of a separate protease

**Fig. 8:**
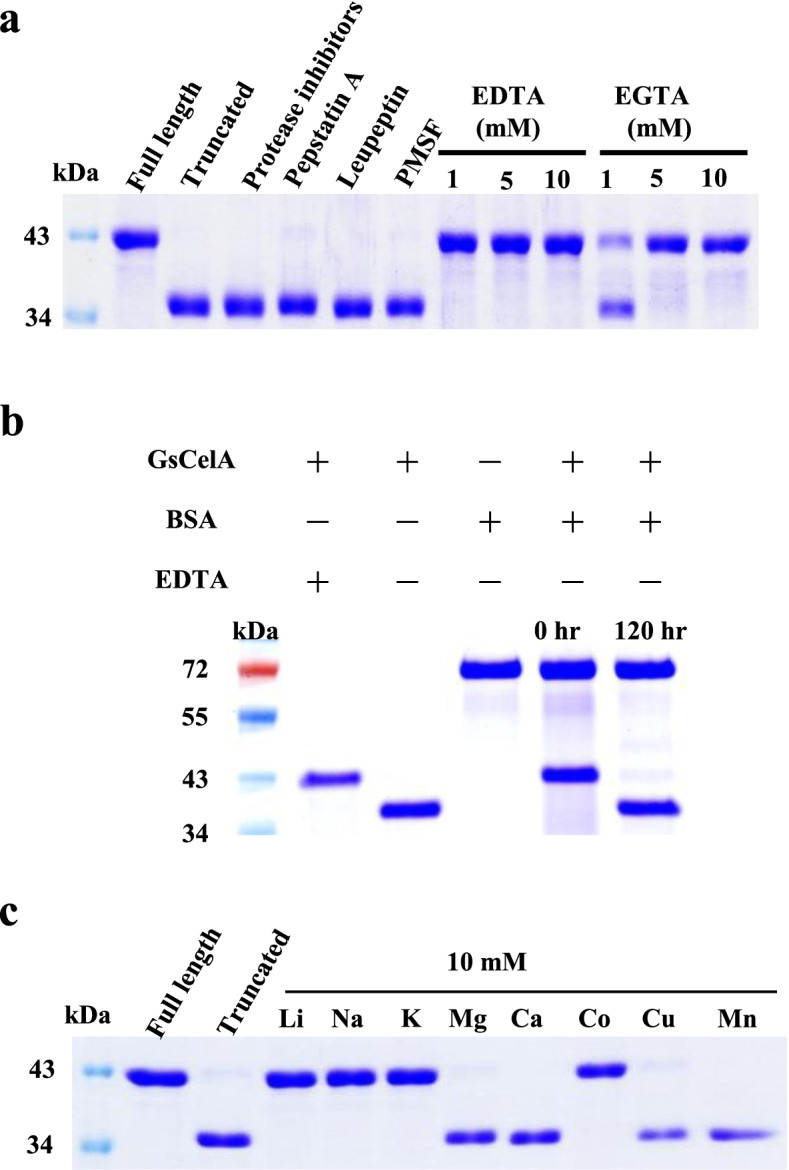
GsCelA truncation process is inhibited by metal chelators but can be recovered with divalent metal ions. **a** FL-GsCelA was incubated in the presence of different protease inhibitors, EDTA and EGTA, at 25 °C for 120 h. EDTA-pretreated GsCelA (i.e., full length) was a negative control. **b** Purified GsCelA does not have general protease activity. GsCelA was co-incubated with BSA at 25 °C for 120 h. Note that BSA did not get degraded yet GsCelA showed self-truncation. Lane 1: protein marker; lane 2: GsCelA pretreated with 10 mM EDTA; lane 3: self-truncated GsCelA; lane 4: BSA only; lane 5 and 6: BSA co-incubated with GsCelA for 0 and 120 h. **c** Effect of presence of divalent ions (i.e., Mg^2+^, Ca^2+^, Cu^2+^, Mn^2+^, or Co^2+^) and monovalent ions (i.e., Li^+^, Na^+^ or K^+^) on recovering GsCelA’s truncation ability. Different metal ions were added to EDTA-pretreated GsCelA to a final concentration of 10 mM, and the incubation was performed at 25 a for 120 h

To investigate whether the self-truncation process could be due to the contamination of a separate protease, we treated highly purified FL-GsCelA with different protease inhibitors and metal chelators, including a commercial protease inhibitors cocktail (EDTA free, from Roche Switzerland), pepstatin A, leupeptin, PMSF, EDTA, and EGTA. Self-truncation still occurred in the presence of these inhibitors, except EDTA and EGTA (Fig. 8a). When purified, FL-GsCelA was incubated with an unrelated protein, bovine serum albumin (BSA), at 25 °C for 120 h, GsCelA still self-truncated, but BSA remained intact (Fig. 8b). Furthermore, a protease assay was also performed with the purified GsCelA using casein as the substrate. In comparison with the metalloprotease thermolysin, the purified GsCelA did not exhibit any detectable protease activity in 48 h at 25 °C (Supplementary Figure 8, Additional file 1).

**Fig. 9:**
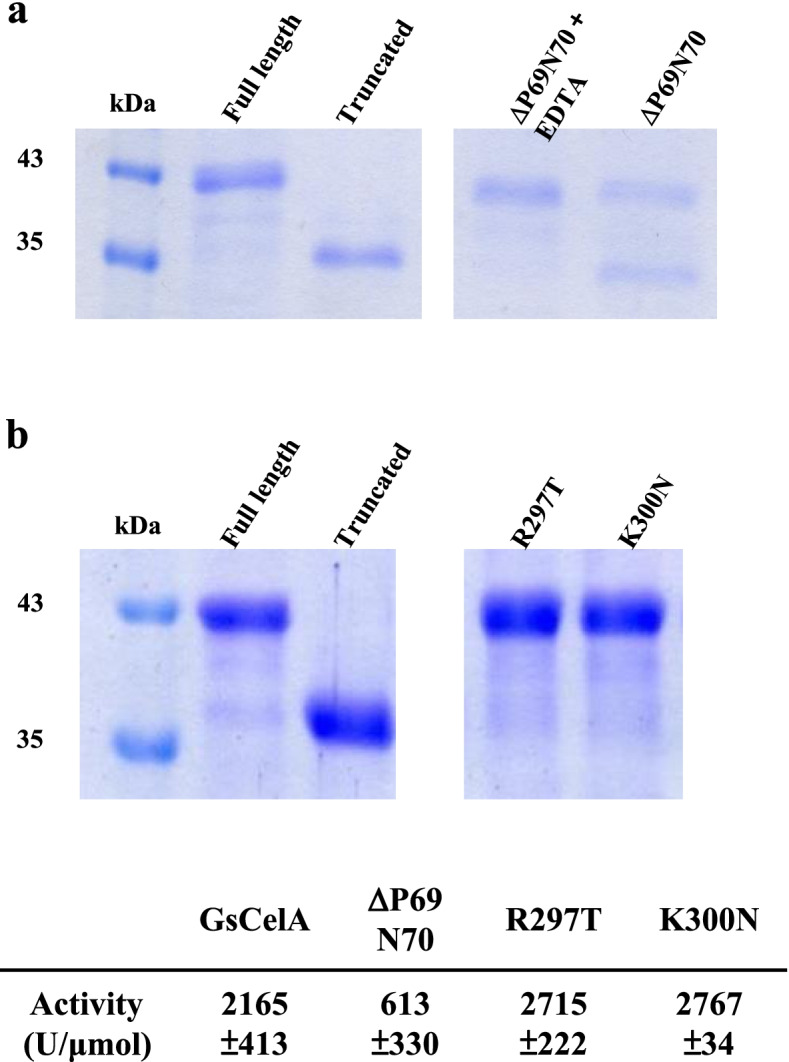
The GsCelA self-truncation process is affected negatively in ΔP69T70, R297T, and K300N mutants. **a** The truncation process was slowed down in the GsCelA mutant with deletion of amino acids P69 and N70 (ΔP69N70). **b** Single-amino-acid substitution R297T or K300N abolishes the self-truncation process. Full-length and truncated GsCelA were used as positive control. The marker lane and protein profiles were cut from the same SDS-PAGE gel

The self-truncation process was observed in both FL-GsCelA and its C-terminal truncated mutants. The self-truncation of FL-GsCelA was affected negatively in a two amino acid-deletion mutant and also in two single-substitution mutants. Amino acids P69 and N70 are located on a loop in the peripheral region of GsCelA [18], and this loop is absent in other GH5 EGs homologues (Supplementary Figure 9, Additional file 1). The deletion of these two amino acids (ΔP69N70) showed reduced self-truncation after 120 h incubation (Fig. 9a) as well as significant reduction of EG activity. Based on sequence alignment analyses, we have also observed that there is a significant sequence difference in the region upstream from the cleavage site A315 among GsCelA and its homologous EGs (Supplementary Figure 9, Additional file 1). To investigate if modification of this region upstream from the cleavage site could also affect the self-truncation process, we have chosen to replace the positively charged amino acid residues R297 and K300 by site-directed mutagenesis. The two single-amino-acid substitution mutations, R297T and K300N, failed to exhibit self-truncation after the same incubation time (Fig. 9b). It is interesting to note that the EG activity was still retained in these two single-amino-acid mutants. Together with the lack of metalloprotease activity in the GsCelA sample and inhibition of truncation by EG substrate CMC and product cellobiose, our results suggest that GsCelA truncation is not likely due to contamination of a separate protease, and it appears to be a self-catalyzing process.

### Divalent metal ions facilitate GsCelA self-truncation

Because FL-GsCelA remained intact after EDTA and EGTA treatment, we wondered whether self-truncation is a metal ion-dependent process. We tested the effect of three monovalent ions (LiCl, NaCl, and KCl) and five divalent ions (MgCl_2_, CaCl_2_, CoCl_2_, CuCl_2_, and MnSO_4_) on self-truncation of EDTA-pretreated FL-GsCelA. The self-truncation process did not appear in the presence of any monovalent ions, i.e., Li^+^, Na^+^, and K^+^, and Co^2+^ (Fig. 8c) but was recovered in the presence of Mg^2+^, Ca^2+^, Cu^2+^, and Mn^2+^ after incubation at 25 °C for 120 h, which suggests that the GsCelA self-truncation process depends on certain types of divalent ions.

### Self-truncation process of GsCelA in Geobacillus

**Fig. 10:**
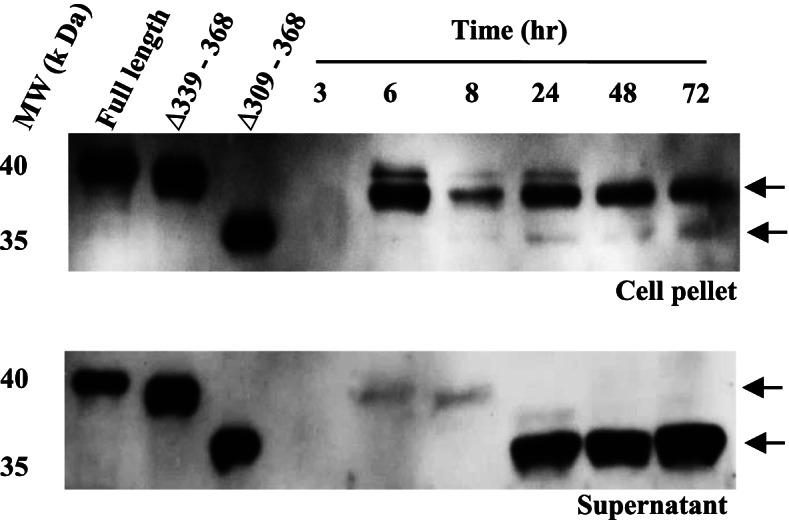
*Geobacillus* sp. 70PC53 secretes native GsCelA in truncated form into culture medium. Identification of native GsCelA using Western blotting with specific antibody against GsCelA. Samples were collected from bacteria pellet and culture medium fractions of *Geobacillus* sp. 70PC53. Recombinant wild-type and mutant enzymes (∆339–368 and ∆309–368) were applied as indicators for full-length and truncated GsCelA, respectively

The optimal culture temperature for the thermophilic bacterium *Geobacillus* 70PC53 is about 50 to 60 °C. To investigate the truncation pattern of native GsCelA in *Geobacillus* 70PC53, the bacterial culture was incubated at 60 °C, and the presence of GsCelA and its truncated products were detected with the Western blotting method using specific antibodies raised against purified GsCelA. The production of apparent FL-GsCelA in *Geobacillus* was observed 6 h after starting a fresh culture. The truncated GsCelA was primarily observed in the culture medium after 24 h of incubation (Fig. 10). These results indicate that *Geobacillus* 70PC53 synthesizes FL-GsCelA in the cells and secretes the truncated form enzyme into the extracellular environment.

### C-terminal region of GsCelA is capable of binding to cellulose

**Fig. 11:**
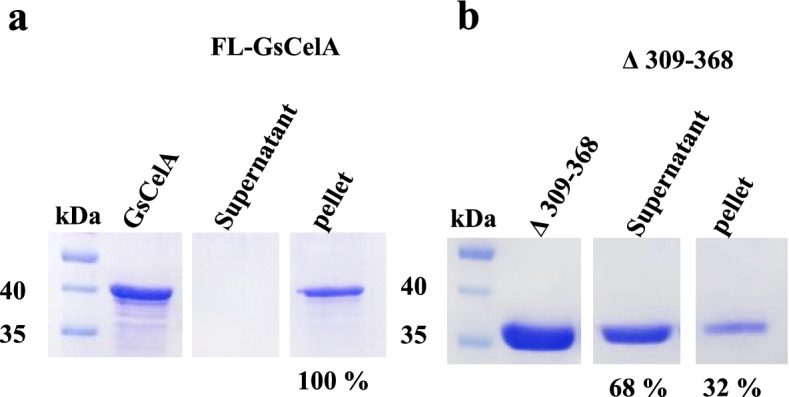
FL-GsCelA is capable of binding to swollen Avicel. **a** FL-GsCelA and **b** ∆309–368 were tested for their ability to bind the swollen Avicel (i.e., amorphous cellulose). Lane 1: purified GsCelA used in this binding assay; lane 2: supernatant collected after test; lane 3: pellet collected at the end of the test. Protein profiles of FL-GsCelA or ∆309–368 in this figure were cut from the same SDS-PAGE gel. The relative amounts of protein bands, i.e., percentage numbers underneath the gel lanes, were estimated from stained gel by using the image processing program ImageJ

To determine the function of C-terminal region in FL-GsCelA, which is removed by the self-truncation process, FL-GsCelA or Δ309–368 was co-incubated with swollen Avicel to investigate how FL-GsCelA interacts with its substrate. Upon incubation with swollen Avicel followed by centrifugation, it was observed that virtually all FL-GsCelA was associated with the pellet, i.e., Avicel (Fig. 11a), indicating that FL-GsCelA was bound to the substrate. In contrast, about two thirds of Δ309–368 truncated proteins were observed in the supernatant (Fig. 11b). Therefore, the presence of the C-terminal region leads to the adsorption of FL-GsCelA by cellulose.

## Discussion

GsCelA is a secreted GH5 family EG from the thermophilic bacterium *Geobacillus* sp. 70PC53 isolated from rice straw compost. Fortuitously, we observed that a smaller protein appeared spontaneously from highly purified recombinant GsCelA after long-term storage at 4 °C, and this smaller protein exhibited enhanced catalytic activity and thermostability. This intriguing phenomenon prompted us to investigate how GsCelA processes this apparent self-truncation.

### GH5 EGs undergo spontaneous truncation

GH5 family is one of the largest groups of the glycoside hydrolase families. GH5 cellulolytic enzymes are found in a wide range of organisms, and most of them are bacterial EGs. All of these EGs share similar TIM-barrel structure with two glutamic acids essential for catalysis. Several secreted EGs from *Bacillus* species contain a CBM3 in their C-terminal region. GsCelA is a typical GH5 EG, and its crystal structure reveals an expected TIM-barrel structure with 308 amino-acid residues (PDB code: 4XZB) [18]. Protein sequence analyses indicated that this EG does not contain any known CBM in its C-terminal region. In our study, we chose three GH5 EGs, GsCelA and BsCel5A in the GH5-2 subfamily, and SqEGV in the GH5-4 subfamily, which are phylogenetically distant (Supplementary Figure 1, Additional file 1), and one GH12 EG (RSC-EG1) for analysis. Both GsCelA and SgEGV do not have a known CBM, whereas BsCel5A has a CBM3. GH12 EG has a β jelly-roll structure [22], which is quite different from the TIM-barrel structure in GH5 EGs. Our results indicated that among the four highly purified EGs, spontaneous truncation was observed with all GH5 enzymes, i.e., GsCelA, SgEGV, and BsCel5A (Fig. 2) but not with GH12 RSC-EG1, which suggests that this truncation process is likely to be associated with GH5 EGs. It is worth noting that all these GH5 EGs are produced by Gram-positive bacteria; however, whether GH5 EGs from other types of organisms have similar properties awaits more studies.

An earlier study showed that a secreted EG from *Bacillus subtilis* is truncated upon prolonged culturing of this bacterium, and this phenomenon is due to the action of a separate protease [11–13, 23]. Furthermore, the *E. coli*-expressed recombinant *Bacillus* EG was cleaved in the periplasmic space of the bacterium by an *E. coli* protease, and the truncated enzyme exhibited higher activity [13]. In our studies, MS analysis confirmed that the freshly and highly purified *E. coli*-expressed recombinant GsCelA is a full-length protein (FL-GsCelA, Supplementary Figure 3b, Additional file 1), which indicates that no additional processing occurs to FL-GsCelA in the *E. coli* expression system. It is important to note that the sequence of FL-GsCelA expressed in *E. coli* is exactly the same as in the GsCelA gene in the *Geobacillus* 70PC53 genome (Supplementary Figure 2, Additional file 1). Furthermore, MS analysis of highly purified GsCelA after long-term storage at 4 °C revealed that only one protein with a length of 315 amino-acid residues is present in the sample (Supplementary Figure 3c, Additional file 1), which indicates that GsCelA truncation is a spontaneous post-translational process without the participation of a separate protein. Similar situation also takes place with the other GH5 EGs tested in this study, i.e., apparent self-truncation in highly purified BsCel5A and SgEGV without the participation of a separate protein.

### GsCelA spontaneous truncation is independent of a separate protease, thus a self-truncation process

Trypsin activation via the protein cleavage by a separate protease is a well-studied case of post-translational process for enzyme maturation. In humans, enteropeptidase hydrolyzes the activation peptide of trypsinogen (i.e., trypsin precursor) between K23 and I24 [24, 25]. In addition, trypsinogen is capable of autoactivation that is sensitive to specific pH and concentration of calcium ion [26]. Because trypsin digests peptide/protein by recognizing lysine (K) or arginine (R) and cuts behind these two amino acids, it is also able to activate trypsinogen via autoactivation by cutting the activation peptide from K23 [27]. Unlike the N-terminal processing of trypsinogen, GsCelA undergoes a C-terminal truncation between K315 and G316 forming a 315-amino-acid-long protein. Furthermore, none of the inhibitors against the cysteine- (such as trypsin), threonine-, or aspartic acid type proteases could suppress the GsCelA self-truncation process (Fig. 8a). On the other hand, divalent metal chelators, EDTA and EGTA, were effective in blocking the truncation process. These chelators can remove metal ions from a protein molecule, and we have indeed observed the presence of a calcium ion in the structure of GsCelA [18]. Although it is not known whether the GsCelA-bound calcium ion is involved in the truncation process, several metal ions were tested for their effect on restoring the self-truncation of GsCelA that has been pretreated with EDTA. Indeed, Ca^2+^ and also several other divalent ions, i.e., Mg^2+^, Mn^2+^, and Cu^2+^, could restore GsCelA self-truncation capability (Fig. 8c). Therefore, it appears that the glycolytic enzyme GsCelA also possesses a calcium-dependent proteolytic activity responsible for the C-terminal self-truncation process. To further rule out that a separate protease could be involved and also to address the question whether the proteolytic activity of GsCelA can digest other proteins, BSA was added to the purified GsCelA preparation. However, GsCelA was still truncated, while BSA remained intact (Fig. 8b). The purified GsCelA has also been tested for potential contamination of a separate protease, yet no detectable metalloprotease activity was detected (Supplementary Figure 8, Additional file 1). Two additional lines of evidence have further ruled out the possibility that the truncation of GsCelA is due to a separate contaminating protease. First, at least three GsCelA mutations impact the truncation negatively (Fig. 9). Second, both the substrate and product, i.e., CMC and cellobiose, respectively, inhibit the truncation process (Supplementary Figure 7, Additional file 1). Although one might argue that mutations and the presence of CMC and cellobiose may alter the conformation of GsCelA rendering the contaminating separate protease incapable of attacking GsCelA, this possibility appears to be unlikely. In addition, GsCelA appears to be synthesized in its natural location, i.e., *Geobacillus*, as a full-length enzyme. It is then truncated upon secretion into the culture medium. Since the truncated GsCelA showed enhanced enzyme activity and thermostability (Fig. 1b, c), and the FL-GsCelA was not cleaved by a separate protease, we suggest that GsCelA be capable of performing its own self-truncation leading to the formation of mature form with enhanced catalytic efficiency. To our knowledge, the self-truncation in GH5 EGs, a non-protease group of glycolytic enzymes, has never been reported before.

### GsCelA self-truncation represents a potential protein self-splicing process

Self-splicing is one of the protein post-translational modifications. VMA1 gene in *Saccharomyces cerevisiae* encodes a 120-kDa vacuolar membrane H (+)-ATPase precursor. The precursor of this protein removes a 454-amino-acid-long internal domain by protein splicing [28], and this internal domain was defined later as an intein. Inteins contain four conservative motifs with specific splicing amino acid residues [29]. The presence of conserved cysteine, serine, or threonine at the C-extein +1 position is required, and the splicing results in the ligation of N-extein and C-extein upon the removal of the intein part. These conservative motifs can promote spontaneous excision of VMA1 precursor into a 70-kDa mature VMA1 [30]. A type III-secreted protein, called nodulation outer protein E1 (NopE1), is secreted by the Gram-negative bacterium *Bradyrhizobium japonicum* and contains an unknown function domain DUF1521 [16]. DUF1521 is 174 amino-acid long with an EF-hand-like motif [16, 31]. The autocleavage of DUF1521 domain in NopE1 is metal ion-dependent, and the cleavage site contains a conserved GD’PHV motif [32]. This domain is sensitive to calcium, and the self-cleavage was observed at a broad range of temperature and pH. In comparison with the two mechanisms described above, GsCelA does not contain any known domains involved in protein self-cleavage. However, because GsCelA self-truncation is temperature-, pH-, and metal ion-dependent (Figs. 7a, b and 8b) and this truncation is also observed in other GH5 EGs tested in this study (Fig. 2), this protein self-truncation could be a new type of protein self-cleavage process specific to GH5 EGs. However, it has to be pointed out, the GsCelA self-truncation process is not a complete self-splicing mechanism because there is no rejoining of peptides after the proteolytic cleavage.

### GsCelA self-truncation is independent of EG activity, and the TIM-barrel structure appears to play a key role

To address the underlying mechanism for this intriguing self-truncation of GsCelA, we further investigated whether this process is dependent on the EG activity or certain structure features. We first mutated the catalytic residues E142 and E231 of GsCelA by amino acid substitution to suppress EG activity. As expected, the glycolytic activities were totally abolished in these active site mutations, yet the self-truncation still took place (Fig. 6), indicating that the protein self-cleavage is independent of EG activity. Next, we generated different GsCelA mutants with progressively shortened C-terminal region. The C-terminal mutants Δ339–368 and Δ309–368 enhanced the enzyme activity without negatively affecting the protein stability (Figs. 3 and 4). Furthermore, deletions of up to 30 amino acid residues at C-terminal region did not suppress the ability of GsCelA to self-truncate (Fig. 5b). However, the mutant Δ298–368 showed decreased enzyme activity and thermostability, probably due to the disruption of the TIM-barrel structure of GsCelA (Fig. 3a). Surprisingly, substitution of K315 at the self-truncation cleavage site in the mutant K315A did not suppress GsCelA self-truncation at all (Fig. 5b). Nevertheless, removal of 10 amino acid residues adjacent to the natural cleavage site in the mutant Δ310–320 slowed down the truncation process (Fig. 5a). However, the truncated enzymes showed different protein lengths from 312 to 319 amino acids (Supplementary Figure 6, Additional file 1), indicating that this peptide sequence contains the target of GsCelA self-truncation. Furthermore, P69 and N70 form an extra small loop peripheral to the main TIM-barrel structure of GsCelA (Supplementary Figure 9, Additional file 1). It has been suggested that the side chain of P69 undergoes a non-polar interaction with I103 and the side chain of N70 stabilizes the loop by hydrogen bonding [18], which reveals the potential role of this loop in stabilizing the TIM-barrel core structure of GsCelA. It is intriguing to note that the mutant with these two amino acids deleted causes the GsCelA self-truncation process to slow down significantly (Fig. 9). Based on these results, we suggest that GsCelA has to retain its TIM-barrel structure to promote its self-truncation. Although the amino acid identity of at the cleavage site does not appear to be important, the peptide sequence surrounding this site plays a role in determining the location of GsCelA truncation. Two single-amino-acid substitution mutations, R297T and K300N, suppress the self-truncation process without impact on the EG activity. The amino acid K300 appears to be highly conserved, being either K or R, as revealed by multiple alignment analysis among 27 GH5 EGs, indicating that this particular amino acid is potentially part of the protease domain involved in the self-truncation process.

### Biological significance of GsCelA truncation

Protein sequence analyses revealed that the C-terminal region of GsCelA has a potential transmembrane helix structure, so this C-terminal region could conceivably interact with the *Geobacillus* 70PC53 cell membrane or hydrophobic regions in cell walls and could be cleaved by GsCelA self-truncation to release the mature GsCelA into the environment (Fig. 10). Furthermore, the C-terminal region of GsCelA is capable of binding to cell walls and to the swollen cellulose (Fig. 11a). Therefore, we suggest that this C-terminal region in GsCelA is likely to play a key role in the docking of this enzyme on *Geobacillus* sp. 70PC53 cell walls, and its catalytic part is then freed by the self-truncation process to attack the substrate with elevated activity and thermostability.

The suppression of self-truncation by the enzymatic product, cellobiose, appears to make sense because there is no need to release more active enzyme by self-truncation once plenty of soluble sugars are around to support the growth of bacterial culture. On the other hand, the suppression by the substrate, CMC, is unexpected, and it could be un-natural due to the presence of a large amount of soluble form of modified cellulose (CMC), a condition that rarely exists in nature. Therefore, the biological significance of this suppression needs to be further investigated in the future.

Bacterial intein-like (BIL) domains, which are similar to intein domain structure but different in sequence features, have a length ranging from 130–155 amino acids with unique sequence motifs [33]. The presence of conserved cysteine, serine, or threonine in the C-terminal end is not obligatory in A-type BILs, whereas these amino acid residues in intein are not preceded by the conserved histidine-asparagine motif in B-type BILs [34, 35]. Moreover, a new conserved domain named putative predator-specific domain 1 (PPS-1) has been discovered in C-type BILs [36]. BILs catalyze protein splicing not only in the ligation of N-terminal and C-terminal peptides, but also in the N-cleavage, C-cleavage, or both N- and C-cleavages [34, 35]. Therefore, the protein self-cleavage mechanism appears to be very diverse among bacterial proteins.

The FL-GsCelA does not contain conserved amino acid motifs observed in inteins or BILs. However, GsCelA displays an obvious C-terminal cleavage phenomenon in both native and recombinant proteins (Figs. 1 and 10). The GsCelA self-truncation is not suppressed in most of mutant enzymes generated by site-directed mutagenesis or C-terminal region deletion, except for mutations at R297 and K300 residues located on the helix α8 in the TIM-barrel part of the enzyme (Figs. 5, 6, and 9) [18]. The same C-terminal self-truncation process was observed in two other GH5 EGs belonging to two different subfamilies, GH5-2 and GH5-4 (i.e., BsCel5A and SgEGV) with known carbohydrate-binding motifs (CBM) that appear to be longer than the C-terminal region of GsCelA (Fig. 2) [19]. Taken together, we suggest that the self-truncation process may exist in at least some GH5 enzymes, and this process is involved in a synergistic/coordinated relationship between two distinct parts of these enzymes, i.e., the TIM-barrel basic structure and the C-terminal CBM.

## Conclusions

We have discovered a novel self-truncation/activation process that appears to be common among GH5 endoglucanases produced by Gram-positive bacteria, and demonstrated that GsCelA can enhance its catalytic activity and thermostability via a novel self-truncation process. This process is independent of EG activity, and it is not caused by a separate proteolytic enzyme and is sensitive to pH, temperature, and the presence of divalent metal ions. The TIM-barrel structure in GsCelA may be essential for this self-truncation capability. Because at least two other EGs but not GH12 EG show the same self-truncation phenomenon, this GsCelA self-truncation is a novel protein self-cleavage process specifically observed in some GH5 EGs. Further investigations are warranted to reveal the detailed mechanisms of this process.

## Methods

### Production of recombinant EGs

The EG GsCelA coding sequence was identified from the *Geobacillus* sp. 70PC53 strain as described previously [17]. BsCel5A was cloned by PCR amplification from *Bacillus subtilis* strain 168 genomic DNA [19]. SgEGV is a secreted EG from *Streptococcus* sp. that was isolated from goat rumen by our team in Tainan, Taiwan. RSC-EG1 was identified from rice straw compost and cloned following a metagenomics approach [20]. The PCR product of EGs coding sequence was cloned into pET-20b (+), which adds a 6-His tag at the C-terminus of the recombinant protein, and the expression vector was transformed into *E. coli* strain Rosetta (DE3). The enzyme production involved using LB medium containing 100 μg/ml ampicillin and 34 μg/ml chloramphenicol. The bacterial culture was incubated with a shaking speed of 200 rpm at 37 °C until the OD_600_ value reached 0.5. After adding 0.5 mM isopropyl-β-D-1-thiogalactopyranoside (IPTG) for inducing the expression of recombinant protein, the incubation continued at 16 °C for 15 h.

### Phylogenetic and DNA sequence analyses

The GsCelA protein sequence was uploaded to the NCBI protein-protein Blast program and was compared to the protein sequence database to identify potential EG homologs (Supplementary table 1, Additional file 1). The software MEGA7 [37] was used to determine protein evolutionary relationships, and a phylogenetic tree was constructed by Neighbor-Joining method. Clustal Omega software (http://www.ebi.ac.uk/Tools/msa/clustalo/) was used for sequence alignment analysis.

### Construction of GsCelA C-terminal variants and mutants

The pET-20b (+) plasmid carrying the FL-GsCelA was used as a template to generate GsCelA variants with a truncated C-terminal region and other mutants. Primers used for these experiments are listed in Supplementary table 2, Additional file 1. Site-directed mutagenesis involved using the QuikChange II Site-Directed Mutagenesis Kit (Agilent Technologies) to generate GsCelA mutants carrying one or multiple mutations. GsCelA mutants with deleted internal sequences were generated by using a modified version of the gene splicing protocol [38]. All PCR amplifications were performed with Phusion polymerase (Thermo Fisher, USA). Briefly, fragments A and B, with 20-bp overlapping ends on each fragment, were generated independently with the primer pairs AF/AR and BF/BR. The first step of overlap extension PCR was performed with equal quantities of fragments A and B under the following PCR conditions: pre-denaturation (98 °C for 30 s), 10-cycle amplifications (98 °C for 10 s, 55 °C for 2 min and 30 s, and 72 °C for 2 min and 30 s), and final extension (72 °C for 1 min). A second PCR was performed with 7.5 μl reaction product and the primer pair AF/BR under the following conditions: pre-denaturation (98 °C for 30 s), 30-cycle amplifications (98 °C for 30 s, 60 °C for 10 s, and 72 °C for 2 min and 30 s), and final extension (72 °C for 2 min).

### Recombinant EG purification

Purification of *E. coli*-expressed EGs was carried out with affinity chromatography using a FPLC work station (ÄKTA purifier, Sweden). The culture medium was centrifuged at 6000×*g* for 20 min at 4 °C, and the supernatant was removed. Cells were re-suspended in 20 mM sodium phosphate buffer (pH 7), then disrupted by using a high-pressure cell disruptor (Constant System TS 2.2kW, UK). The cell debris was removed by centrifugation at 8000×*g* for 20 min at 4 °C, and the supernatant was filtered through a 0.45-μm pore-size membrane filter. The filtered supernatant was applied to a 1-ml His-Trap FF crude affinity chromatography column. The mobile phase was 20 mM sodium phosphate buffer (pH 7) with 0.5 M sodium chloride and 20 mM imidazole. Recombinant EG was eluted from the column with a linear gradient of elution buffer containing 20 to 500 mM imidazole, 20 mM sodium phosphate (pH 7), and 0.5 M sodium chloride.

### SDS-PAGE analysis and zymography

The molecular weight and enzyme activity of recombinant GsCelA were determined by SDS–polyacrylamide gel electrophoresis. Recombinant GsCelA was denatured by heating at 100 °C for 10 min with 5× sample buffer (250 mM Tris-HCl, 10% SDS, 30% glycerol, 25% β-mercaptoethanol and 0.05% bromophenol blue, pH 6.8), and samples were loaded on 12% SDS-PAGE gel containing 0.2% (w/v) carboxymethyl cellulose (CMC). For molecular weight determination, SDS-PAGE gel was stained with Coomassie brilliant blue, and protein bands of interest were compared with the molecular weight standards. To analyze the EG activity by zymography, the gel was washed three times (30 min each) with 10 mM Tris-HCl (pH 8) containing 1% Triton X-100 to renature GsCelA. Afterwards, the gel was incubated at 65 °C in 50 mM sodium acetate buffer (pH 5) for 30 min, followed by staining with 2% Congo red for 20 min and destained with 1 M sodium chloride. GsCelA activity was observed on the gel as clear zone against the red background.

### N-terminal sequencing

FL-GsCelA and truncated GsCelA were visualized by SDS-PAGE analysis, and their respective protein bands were transferred to PVDF membrane. Protein samples were cut from PVDF membranes and sent to Mission Biotech (Taipei) for protein N-terminal sequencing. The sequencing was based on the Edman Degradation method and carried out with the Applied Biosystems LC 494 Procise Protein Sequencing System.

### Mass spectrometry analysis

Peptide sequences and precise molecular weights of purified GsCelA and its truncated form were identified by mass spectrometry (MS). For the sample preparation, the protein bands were collected by cutting after SDS-PAGE analysis. The gel slices soaked in a 1.5-mL Eppendorf tube in 25 mM ammonium bicarbonate (pH 8.5) were treated with 100 μl of 50 mM dithioerythritol to reduce and break disulfide bonds, then with 100 μl of 100 mM iodoacetamide to alkylate-free sulfhydryl groups of cysteine. The gel slices were then homogenized in a tube with a plastic pestle followed by treatment with 0.01 μg/μl trypsin or chymotrypsin (i.e., in-gel digestion) at 37 °C for at least 16 h. After centrifugation at 10,000×*g* for 1 min, peptides in the supernatant were desalted by using Zip Tip (Merck, Germany) and dried by vacuum centrifugation (Eppendorf, Germany). For molecular weight determination, samples of highly purified FL-GsCelA and truncated GsCelA proteins were dried by using a SpeedVac vacuum concentrator. All MS samples were analyzed by using Thermo Orbitrap Elite Mass Spectrometer (USA) and Mascot software; the service was provided by the Proteomics Mass Spectrometry Common Facility in the Institute of Biological Chemistry, Academia Sinica, Taiwan.

### Endoglucanase assays with CMC and swollen Avicel

Swollen Avicel (phosphoric acid-swollen Avicel [PASA]) was prepared from Avicel (Sigma). About 0.2 g Avicel suspended in 0.6 ml dH_2_O was added to 10 ml ice-cold 86.2% H_3_PO_4_ slowly and mixed evenly. The Avicel mixture turned transparent within several minutes and was allowed to stand on ice with occasional stirring for 1 h. Forty ml ice-cold dH_2_O were added to the Avicel mixture with vigorous stirring, and the supernatant was removed after centrifugation at 5000×*g* and 4 °C for 20 min. The pellet was re-suspended in ice-cold dH_2_O and centrifuged to remove the supernatant, and the pellet was washed again four times with dH_2_O. Half milliliters of 2 M Na_2_CO_3_ was added to neutralize the residual phosphoric acid, and the Avicel pellet was suspended in 45 ml ice-cold dH_2_O. The re-suspended Avicel was centrifuged at 5000×*g* and 4 °C for 20 min, and the pellet was re-suspended in dH_2_O until pH 6–7. Enzyme activity was determined by measuring reducing sugar concentration with the dinitrosalicylic acid (DNS) method [39]. The reaction solution containing 200 μl of 1% CMC in 50 mM sodium acetate (pH 5) and 2 μg of recombinant GsCelA was incubated for 5 min at different temperatures. After hydrolysis, the reaction mixture was incubated at 100 °C with 200 μl DNS. The reducing sugar concentration was determined by measuring the absorbance at 550 nm. One unit of endoglucanase activity corresponds to 1 μmol reducing sugar generated per min. A standard curve with glucose was established for estimating the reducing sugar equivalents.

### Circular dichroism (CD) spectroscopy analysis

Protein samples were prepared at 10 μM in 20 mM sodium phosphate buffer (pH 7). Far-UV CD spectra (190–260 nm) were recorded on a Jasco-815 spectrometer (Jasco, Japan) using a 1-mm quartz cuvette. Data collection parameters were set to a scan rate of 50 nm/min, response time 4 s, sensitivity 100 mdeg, accumulation 10, heating rate 1 /min, and 60-s delay time for spectrum collection. All thermal unfolding experiments measuring melting temperatures were monitored at 222 nm.

### Conditions for GsCelA self-truncation

To determine the optimal temperature, the enzyme reaction was performed for 5 min at a temperature from 45 to 80 °C. To determine the thermostability, the reaction solution was incubated at 45 to 80 °C for 4 h, and the residual endoglucanase activity was measured at 60 °C with 1% CMC in 50 mM sodium acetate buffer (pH 5). For the pH effect, GsCelA was incubated in various solutions including 50 mM succinate buffer (pH 5 and 6), 50 mM sodium phosphate (pH 6 and 7), and 50 mM Tris-HCl (pH 7 and 8) for 24 h. For the temperature effect, GsCelA was incubated in sodium phosphate buffers at 4 to 30 °C for 24 h. Self-truncation was observed by SDS-PAGE every 24 h. ImageJ was used to quantify FL-GsCelA and truncated GsCelA profiles on SDS-PAGE gel image to calculate the truncation rate.

### Effect of protease inhibitors on GsCelA self-truncation

Six kinds of protease inhibitors were used to investigate whether the GsCelA self-truncation process was due to conventional protease activity: 1× cOmplete EDTA-free protease inhibitor cocktail (Roche, Switzerland), 10 μM pepstatin A (Sigma, USA), 100 μM leupeptin (Sigma, USA), and 1 mM PMSF (ACROS, Belgium), 10 mM EDTA (Sigma, USA), and 10 mM EGTA (Sigma, USA). These inhibitors were incubated with purified GsCelA, and self-truncation was determined every 24 h by SDS-PAGE analysis.

### Protease activity assay

The protease activity assay [40] was determined by using 50 μg purified GsCelA and with 0.6% casein as substrate. The reaction solution contained 300 μl of 1% casein in 50 mM sodium phosphate (pH 7), and 200 μl of different concentrations of thermolysin (Sigma-Aldrich) was used as positive control. The solution was incubated at 25 °C for different reaction times, and the reaction was stopped by adding 50% trichloroacetic. After centrifugation at 12,000×*g* for 10 min, the optical density (OD) of supernatants was measured at 280 nm. One unit of protease activity corresponds to 1 μM of tyrosine per min under the assay conditions.

### Effect of metal ions on GsCelA self-truncation

GsCelA was pretreated with 10 mM EDTA in 50 mM sodium phosphate buffer (pH 7) for 2 h at 4 °C, and the solution was dialyzed with 20 mM sodium phosphate buffer for 3 h at 4 °C to remove EDTA. Then, the pretreated GsCelA was incubated in the buffer containing 10 mM monovalent (from LiCl, NaCl, KCl) or divalent metal ions (from MgCl_2_, CaCl_2_, CoCl_2_, CuCl_2_, and MnSO_4_) for 24 h, and the self-truncation was verified by SDS-PAGE analysis.

### Native GsCelA extraction from Geobacillus

*Geobucillus* sp. 70PC53 was precultured on minimal requirement (MR) plate containing 1% CMC/glucose at 50 °C for 24 h. One single colony was transferred to MR medium containing 1 % CMC, and the culture was incubated at 50 °C overnight. The MR medium consisted of 1.4 g (NH_4_)_2_SO_4_, 2.0 g KH_2_PO_4_, 0.34 g CaCl_2_·2H_2_O, 0.30 g MgSO_4_·7H_2_O, 5 mg FeSO_4_·7H_2_O, 1.6 mg MnSO_4_·H_2_O, 1.4 mg ZnSO_4_·7H_2_O, and 2.0 mg CoCl_2_·6H_2_O per liter. The cell density was determined by OD_600_ absorbance and measured every 3 h for the first 12 h, and then 24 h to the fifth day. Cell pellet and culture medium were separated by centrifugation. *Geobacillus* cell pellet was pretreated with 1 mg/ml lysozyme for 1 h at room temperature, and the pellet (cytoplasm) was separated from the supernatant (peptidoglycan) by centrifugation. The pellet was re-suspended in 20 mM sodium phosphate (pH 7), and the crude protein was extracted by sonication. The culture medium was concentrated and buffer exchanged using a 10,000 MWCO Vivaspin (GE).

### Western blot analysis for GsCelA self-truncation

Specific anti-GsCelA antibodies were generated by using recombinant FL-GsCelA produced by *E. coli*. Anti-GsCelA antibodies are able to recognize FL-GsCelA and truncated GsCelA. The protein extraction was separated by SDS-PAGE method and transferred to a PVDF membrane. For Western blot, PVDF membrane was immerged in TBS buffer (0.2 M Tris base and 1.5 M NaCl) containing 5 % slim milk for 1 h, and then washed three time (15 min for each time) with TBST buffer (TBS buffer and 0.1 % tween 20). The primary antibody and the secondary HRP-conjugated antibody were diluted with TBST buffer containing 5 % slim milk. The membrane was incubated with primary antibodies at 4 °C, overnight. The membrane was then washed three times with TBST, incubated with secondary HRP-conjugated antibody for 1 h at room temperature and washed with TBST for three times. For the detection, the membrane was incubated with ECL substrate (Clarity Max Western ECL substrate, Bio-Rad) for 1 min at room temperature and the image was revealed on an X-ray film.

### Substrate binding test for GsCelA and mutants

One hundred microliters of FL-GsCelA or ∆309-368-GsCelA (0.3 mg/ml) was incubated with 1% acid-swollen Avicel at 4 °C for 1 h. The mixture was centrifuged at 10,000×*g* for 10 min and the supernatant was collected. The pellet was washed once with 100 μl sodium phosphate buffer (20 mM, pH 7). After centrifugation, the supernatant was removed and the pellet was re-suspended in 100 μl sodium phosphate buffer. Fifteen microliters of the supernatant collected from the first centrifugation and the washed pellet were used for SDS-PAGE analysis.

## Supplementary Information

**Additional file 1: Supplementary Figure 1.** Phylogenetic tree of GH5 endoglucanases, including FL-GsCelA. SgEGV and BsCel5A. The three phylogenetically distant GH5 EGs, i.e. GsCelA, SgEGV and BsCel5A, exhibit the similar self-truncation process. **Supplementary Figure 2.** Verification of GsCelA sequence in the genomic DNA of *Geobacillus* sp. 70PC53. Amplification of full length GsCelA gene from *Geobacillus* sp. 70PC53 (GsCelA PCR) and alignment with celA open reading frame (*G*. 70PC53 genome) demonstrated C-terminal region existence in the genome of *Geobacillus* sp. 70PC53. The amino acid K315 near by the cleavage site is indicated in red box. Primers used in PCR amplification are indicated by arrows. **Supplementary Figure 3.** Mass spectrometry identification of FL-GsCelA and truncated GsCelA. (a) Result of N-terminal sequencing (in black box) and LC-MS-MS analysis (in red). (b) The molecular weight of FL-GsCelA is 42140 Da. (c) The molecular weight of truncated GsCelA is 35491 Da, which suggests that the self-truncation point is between K315 and G316. **Supplementary Figure 4.** BsCel5A and SgEGV truncated form MW as detected by LC-MS-MS. (a) BsCel5A truncated form MW is 33781 Daltons. (b) SgEGV truncated form MW is about 42000 Daltons. **Supplementary Figure 5.** Comparison of T_50_ (a) and TA_50_ (b) between FL-GsCelA and ∆309-368. T_50_ is similar between FL-GsCelA and ∆309-368, but TA_50_ is higher for ∆309-368 than FL-GsCelA. **Supplementary Figure 6.** Mass spectrometry analysis of self-truncation products of GsCelA mutant Δ310-320. **Supplementary Figure 7.** GsCelA self-truncation was suppressed by CMC and cellobiose. FL-GsCelA was incubated with CMC or cellobiose at 25 °C for 120 h and analysis with 10 % SDS-PAGE gel. **Supplementary Figure 8.** Metalloprotease activity assay of purified GsCelA. Protease activity was detected by using 0.6 % casein as substrate and thermolysin as positive control. One protease unit is defined as 1 μM of tyrosine released per minute. **Supplementary Figure 9.** Sequence alignment analysis between GsCelA and other GH5 EGs with a CBM domain. Based on protein sequence alignment, two amino acids, P69 and N70, are unique in GsCelA. R297 and K300 are two positive charge amino acids near the A315 truncated site. **Supplementary Table 1.** List of GH family 5 proteins mentioned in the phylogenetic tree. **Supplementary Table 2.** List of primers used for construction of point mutations and truncation form mutants.

## Data Availability

All data generated or analyzed during this study are included in this published article and its supplementary information files.

## Abbreviations

EDTA: Ethylene diamine tetraacetic
EGTA: Ethylene glycol tetraacetic acid
PVDF: Polyvinylidene difluoride
DNS: 3,5-Dinitrosalicylic acid
CD: Circular dichroism
IPTG: Isopropyl-β-D-1-thiogalactopyranoside
