# Supplementary material for: A unique self-truncation of bacterial GH5 endoglucanases leads to enhanced activity and thermostability
Source: BMC Biol. 2022 Jun 9;20:137. doi: 10.1186/s12915-022-01334-y (PMC9185962; doi:10.1186/s12915-022-01334-y)
Supplement: Supplementary file 1 — Additional file 1: Supplementary Figure 1. Phylogenetic tree of GH5 endoglucanases, including FL-GsCelA. SgEGV and BsCel5A. The three phylogenetically distant GH5 EGs, i.e. GsCelA, SgEGV and BsCel5A, exhibit the similar self-truncation process. Supplementary Figure 2. Verification of GsCelA sequence in the genomic DNA of Geobacillus sp. 70PC53. Amplification of full length GsCelA gene from Geobacillus sp. 70PC53 (GsCelA PCR) and alignment with celA open reading frame (G. 70PC53 genome) demonstrated C-terminal region existence in the genome of Geobacillus sp. 70PC53. The amino acid K315 near by the cleavage site is indicated in red box. Primers used in PCR amplification are indicated by arrows. Supplementary Figure 3. Mass spectrometry identification of FL-GsCelA and truncated GsCelA. (a) Result of N-terminal sequencing (in black box) and LC-MS-MS analysis (in red). (b) The molecular weight of FL-GsCelA is 42140 Da. (c) The molecular weight of truncated GsCelA is 35491 Da, which suggests that the self-truncation point is between K315 and G316. Supplementary Figure 4. BsCel5A and SgEGV truncated form MW as detected by LC-MS-MS. (a) BsCel5A truncated form MW is 33781 Daltons. (b) SgEGV truncated form MW is about 42000 Daltons. Supplementary Figure 5. Comparison of T50 (a) and TA50 (b) between FL-GsCelA and ∆309-368. T50 is similar between FL-GsCelA and ∆309-368, but TA50 is higher for ∆309-368 than FL-GsCelA. Supplementary Figure 6. Mass spectrometry analysis of self-truncation products of GsCelA mutant Δ310-320. Supplementary Figure 7. GsCelA self-truncation was suppressed by CMC and cellobiose. FL-GsCelA was incubated with CMC or cellobiose at 25 °C for 120 h and analysis with 10 % SDS-PAGE gel. Supplementary Figure 8. Metalloprotease activity assay of purified GsCelA. Protease activity was detected by using 0.6 % casein as substrate and thermolysin as positive control. One protease unit is defined as 1 μM of tyrosine released per minute. Supplementary Figure 9. Sequ [file 12915_2022_1334_MOESM1_ESM.pptx]

## Slide 1
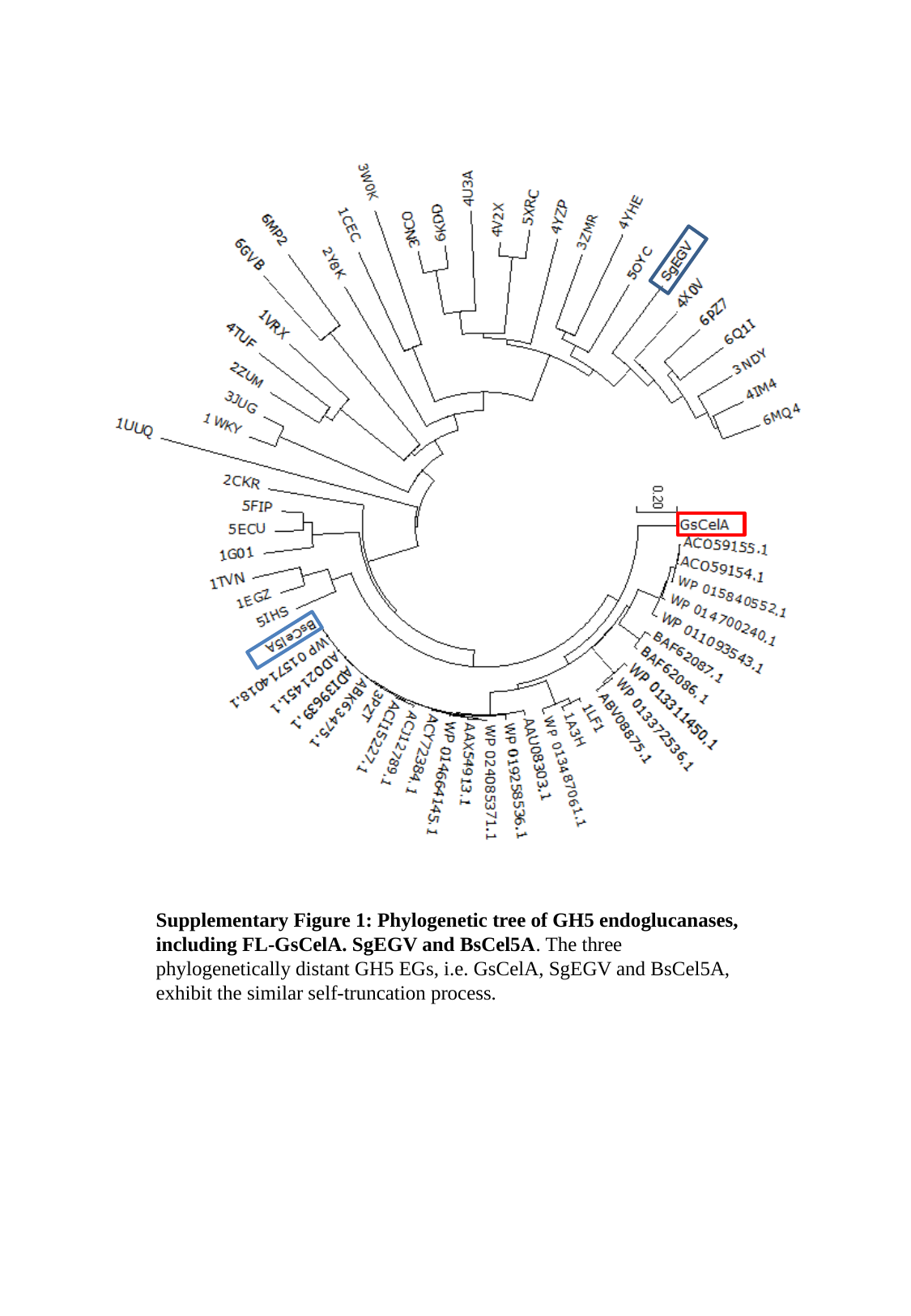

Supplementary Figure 1: Phylogenetic tree of GH5 endoglucanases, including FL-GsCelA. SgEGV and BsCel5A. The three phylogenetically distant GH5 EGs, i.e. GsCelA, SgEGV and BsCel5A, exhibit the similar self-truncation process.

## Slide 2
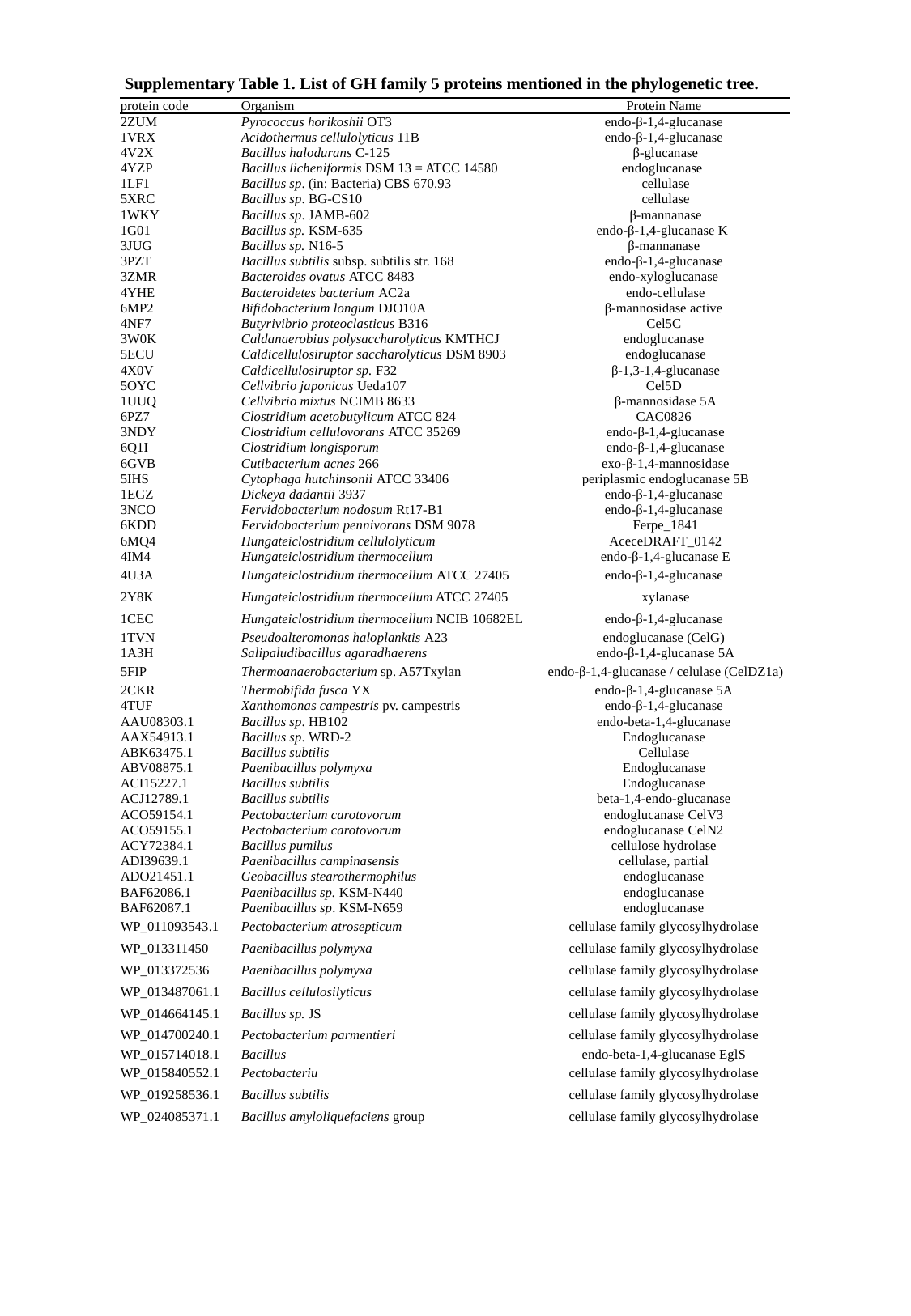

Supplementary Table 1. List of GH family 5 proteins mentioned in the phylogenetic tree.
| protein code | Organism | Protein Name |
| --- | --- | --- |
| 2ZUM | Pyrococcus horikoshii OT3 | endo-β-1,4-glucanase |
| 1VRX | Acidothermus cellulolyticus 11B | endo-β-1,4-glucanase |
| 4V2X | Bacillus halodurans C-125 | β-glucanase |
| 4YZP | Bacillus licheniformis DSM 13 = ATCC 14580 | endoglucanase |
| 1LF1 | Bacillus sp. (in: Bacteria) CBS 670.93 | cellulase |
| 5XRC | Bacillus sp. BG-CS10 | cellulase |
| 1WKY | Bacillus sp. JAMB-602 | β-mannanase |
| 1G01 | Bacillus sp. KSM-635 | endo-β-1,4-glucanase K |
| 3JUG | Bacillus sp. N16-5 | β-mannanase |
| 3PZT | Bacillus subtilis subsp. subtilis str. 168 | endo-β-1,4-glucanase |
| 3ZMR | Bacteroides ovatus ATCC 8483 | endo-xyloglucanase |
| 4YHE | Bacteroidetes bacterium AC2a | endo-cellulase |
| 6MP2 | Bifidobacterium longum DJO10A | β-mannosidase active |
| 4NF7 | Butyrivibrio proteoclasticus B316 | Cel5C |
| 3W0K | Caldanaerobius polysaccharolyticus KMTHCJ | endoglucanase |
| 5ECU | Caldicellulosiruptor saccharolyticus DSM 8903 | endoglucanase |
| 4X0V | Caldicellulosiruptor sp. F32 | β-1,3-1,4-glucanase |
| 5OYC | Cellvibrio japonicus Ueda107 | Cel5D |
| 1UUQ | Cellvibrio mixtus NCIMB 8633 | β-mannosidase 5A |
| 6PZ7 | Clostridium acetobutylicum ATCC 824 | CAC0826 |
| 3NDY | Clostridium cellulovorans ATCC 35269 | endo-β-1,4-glucanase |
| 6Q1I | Clostridium longisporum | endo-β-1,4-glucanase |
| 6GVB | Cutibacterium acnes 266 | exo-β-1,4-mannosidase |
| 5IHS | Cytophaga hutchinsonii ATCC 33406 | periplasmic endoglucanase 5B |
| 1EGZ | Dickeya dadantii 3937 | endo-β-1,4-glucanase |
| 3NCO | Fervidobacterium nodosum Rt17-B1 | endo-β-1,4-glucanase |
| 6KDD | Fervidobacterium pennivorans DSM 9078 | Ferpe\_1841 |
| 6MQ4 | Hungateiclostridium cellulolyticum | AceceDRAFT\_0142 |
| 4IM4 | Hungateiclostridium thermocellum | endo-β-1,4-glucanase E |
| 4U3A | Hungateiclostridium thermocellum ATCC 27405 | endo-β-1,4-glucanase |
| 2Y8K | Hungateiclostridium thermocellum ATCC 27405 | xylanase |
| 1CEC | Hungateiclostridium thermocellum NCIB 10682EL | endo-β-1,4-glucanase |
| 1TVN | Pseudoalteromonas haloplanktis A23 | endoglucanase (CelG) |
| 1A3H | Salipaludibacillus agaradhaerens | endo-β-1,4-glucanase 5A |
| 5FIP | Thermoanaerobacterium sp. A57Txylan | endo-β-1,4-glucanase / celulase (CelDZ1a) |
| 2CKR | Thermobifida fusca YX | endo-β-1,4-glucanase 5A |
| 4TUF | Xanthomonas campestris pv. campestris | endo-β-1,4-glucanase |
| AAU08303.1 | Bacillus sp. HB102 | endo-beta-1,4-glucanase |
| AAX54913.1 | Bacillus sp. WRD-2 | Endoglucanase |
| ABK63475.1 | Bacillus subtilis | Cellulase |
| ABV08875.1 | Paenibacillus polymyxa | Endoglucanase |
| ACI15227.1 | Bacillus subtilis | Endoglucanase |
| ACJ12789.1 | Bacillus subtilis | beta-1,4-endo-glucanase |
| ACO59154.1 | Pectobacterium carotovorum | endoglucanase CelV3 |
| ACO59155.1 | Pectobacterium carotovorum | endoglucanase CelN2 |
| ACY72384.1 | Bacillus pumilus | cellulose hydrolase |
| ADI39639.1 | Paenibacillus campinasensis | cellulase, partial |
| ADO21451.1 | Geobacillus stearothermophilus | endoglucanase |
| BAF62086.1 | Paenibacillus sp. KSM-N440 | endoglucanase |
| BAF62087.1 | Paenibacillus sp. KSM-N659 | endoglucanase |
| WP\_011093543.1 | Pectobacterium atrosepticum | cellulase family glycosylhydrolase |
| WP\_013311450 | Paenibacillus polymyxa | cellulase family glycosylhydrolase |
| WP\_013372536 | Paenibacillus polymyxa | cellulase family glycosylhydrolase |
| WP\_013487061.1 | Bacillus cellulosilyticus | cellulase family glycosylhydrolase |
| WP\_014664145.1 | Bacillus sp. JS | cellulase family glycosylhydrolase |
| WP\_014700240.1 | Pectobacterium parmentieri | cellulase family glycosylhydrolase |
| WP\_015714018.1 | Bacillus | endo-beta-1,4-glucanase EglS |
| WP\_015840552.1 | Pectobacteriu | cellulase family glycosylhydrolase |
| WP\_019258536.1 | Bacillus subtilis | cellulase family glycosylhydrolase |
| WP\_024085371.1 | Bacillus amyloliquefaciens group | cellulase family glycosylhydrolase |

## Slide 3
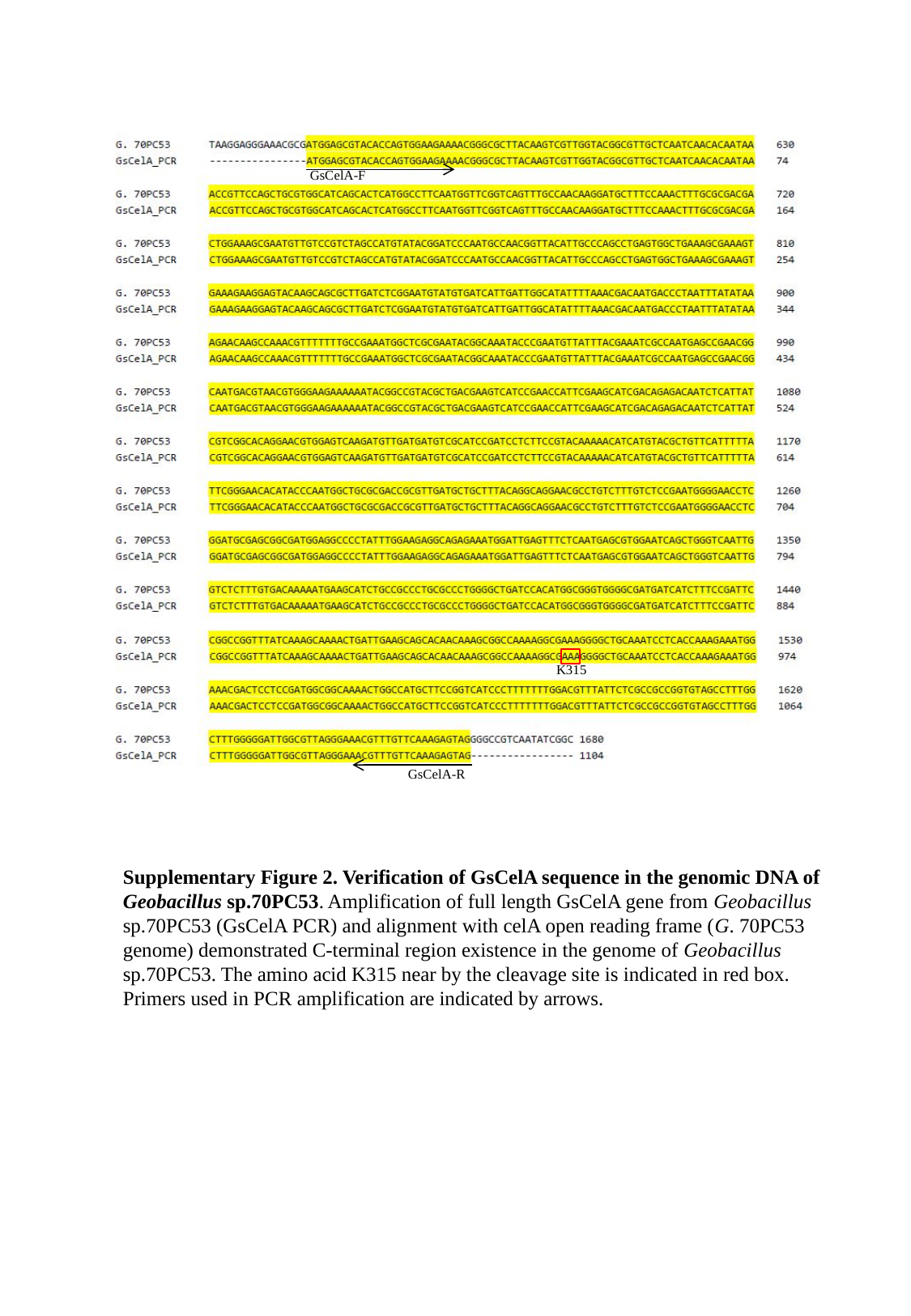

GsCelA-F
K315
GsCelA-R
Supplementary Figure 2. Verification of GsCelA sequence in the genomic DNA of Geobacillus sp.70PC53. Amplification of full length GsCelA gene from Geobacillus sp.70PC53 (GsCelA PCR) and alignment with celA open reading frame (G. 70PC53 genome) demonstrated C-terminal region existence in the genome of Geobacillus sp.70PC53. The amino acid K315 near by the cleavage site is indicated in red box. Primers used in PCR amplification are indicated by arrows.

## Slide 4
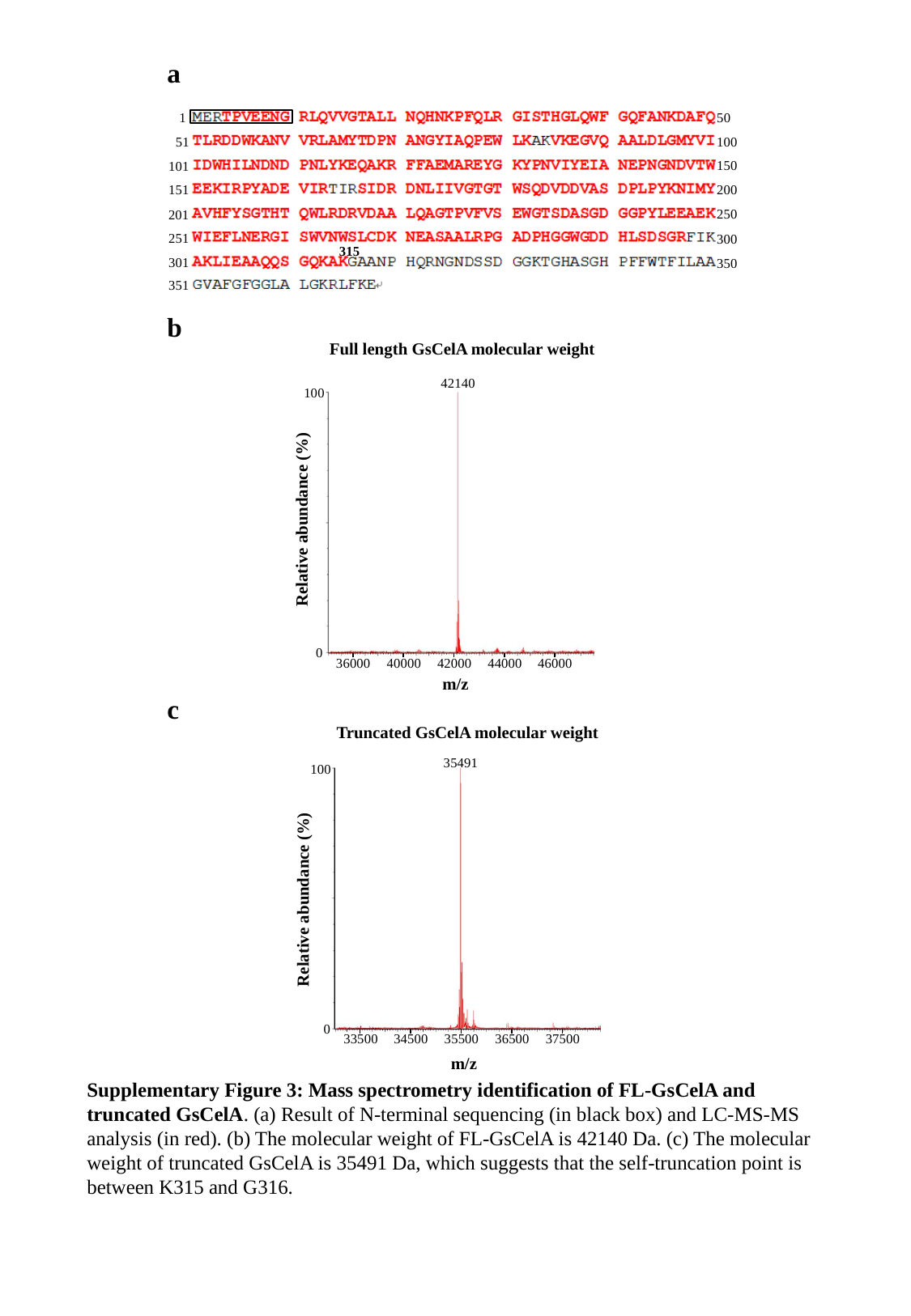

a
1
50
100
51
150
101
151
200
250
201
251
300
301
350
351
315
b
Full length GsCelA molecular weight
42140
100
0
42000
46000
36000
40000
44000
m/z
Relative abundance (%)
c
Truncated GsCelA molecular weight
35491
100
0
37500
33500
34500
35500
36500
Relative abundance (%)
m/z
Supplementary Figure 3: Mass spectrometry identification of FL-GsCelA and truncated GsCelA. (a) Result of N-terminal sequencing (in black box) and LC-MS-MS analysis (in red). (b) The molecular weight of FL-GsCelA is 42140 Da. (c) The molecular weight of truncated GsCelA is 35491 Da, which suggests that the self-truncation point is between K315 and G316.

## Slide 5
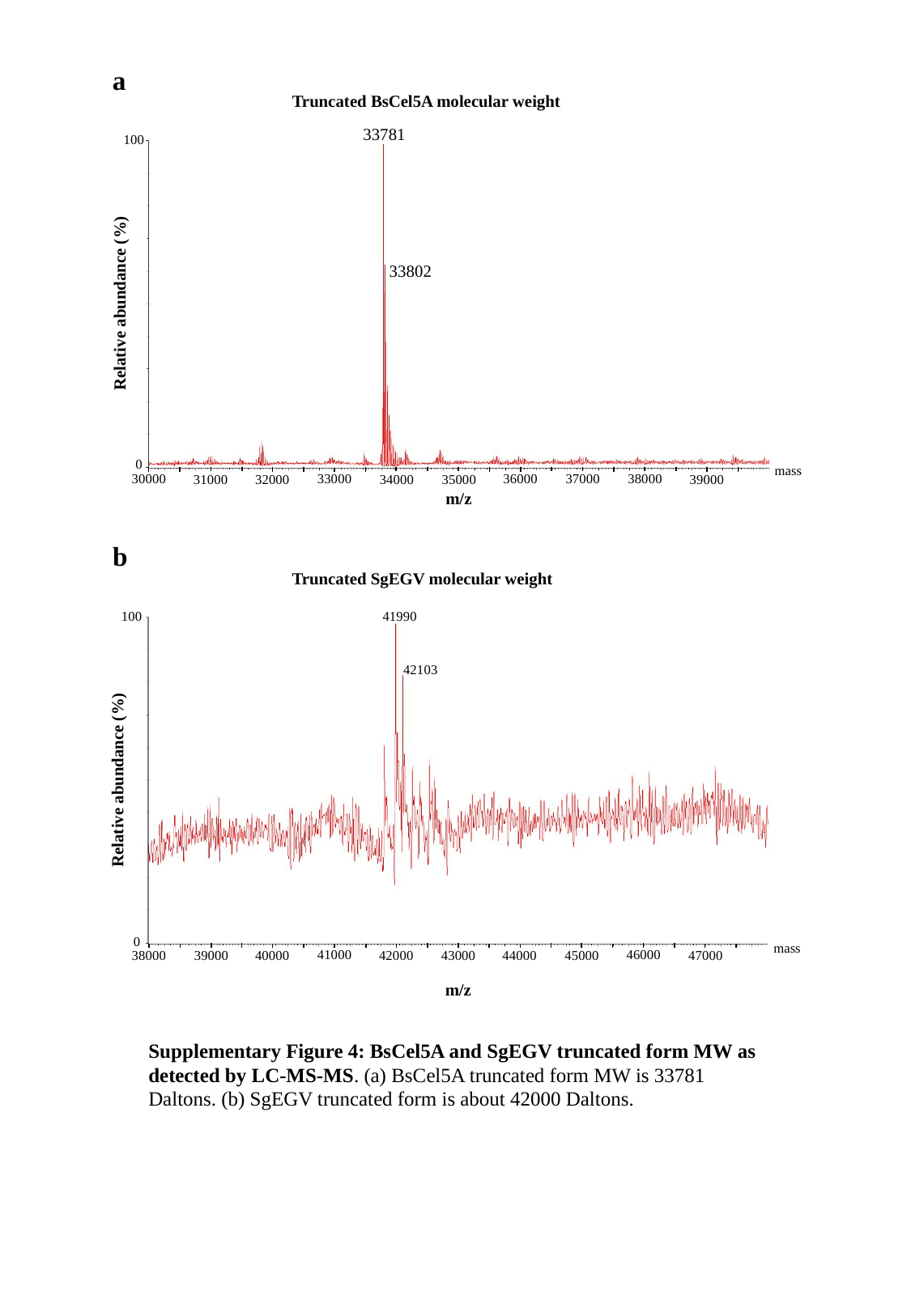

a
Truncated BsCel5A molecular weight
33781
33802
Relative abundance (%)
100
0
mass
36000
33000
30000
37000
38000
34000
35000
31000
32000
39000
m/z
b
Truncated SgEGV molecular weight
41990
42103
100
Relative abundance (%)
0
mass
46000
41000
38000
47000
45000
39000
40000
42000
43000
44000
m/z
Supplementary Figure 4: BsCel5A and SgEGV truncated form MW as detected by LC-MS-MS. (a) BsCel5A truncated form MW is 33781 Daltons. (b) SgEGV truncated form is about 42000 Daltons.

## Slide 6
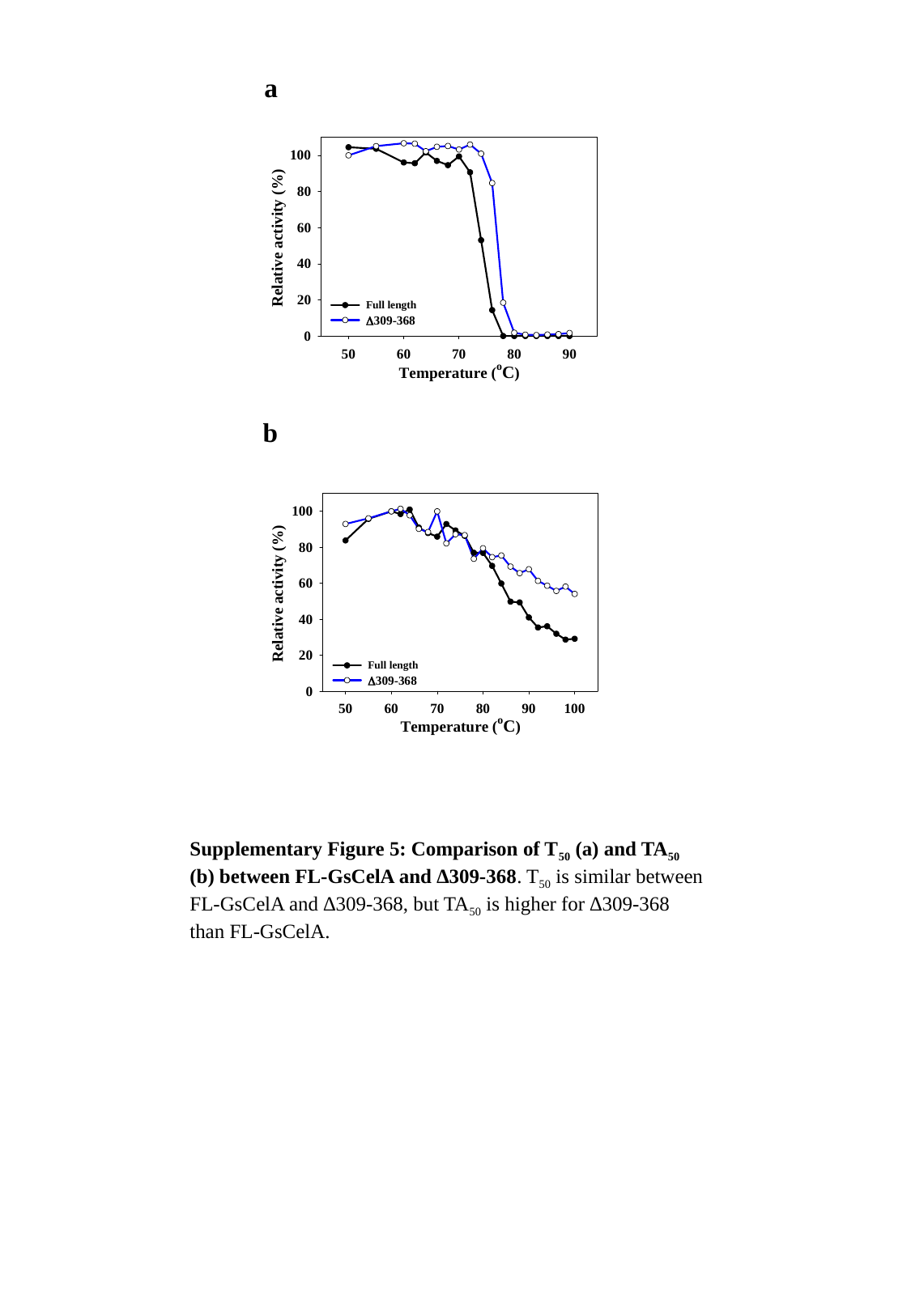

a
b
Supplementary Figure 5: Comparison of T50 (a) and TA50 (b) between FL-GsCelA and ∆309-368. T50 is similar between FL-GsCelA and ∆309-368, but TA50 is higher for ∆309-368 than FL-GsCelA.

## Slide 7
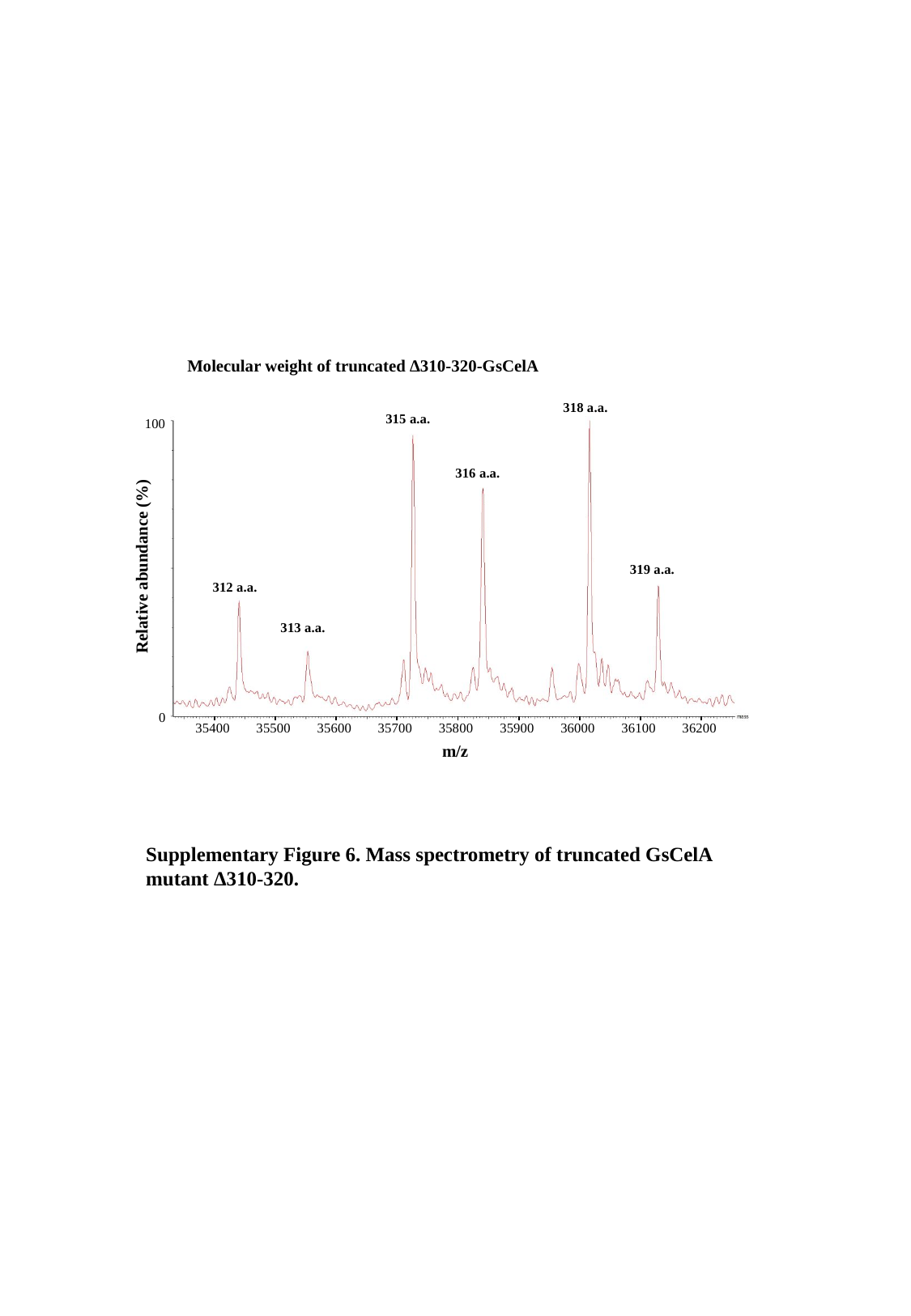

Molecular weight of truncated ∆310-320-GsCelA
318 a.a.
315 a.a.
316 a.a.
319 a.a.
312 a.a.
313 a.a.
100
0
35800
36000
35400
35500
35600
35700
35900
36100
36200
Relative abundance (%)
m/z
Supplementary Figure 6. Mass spectrometry of truncated GsCelA mutant Δ310-320.

## Slide 8
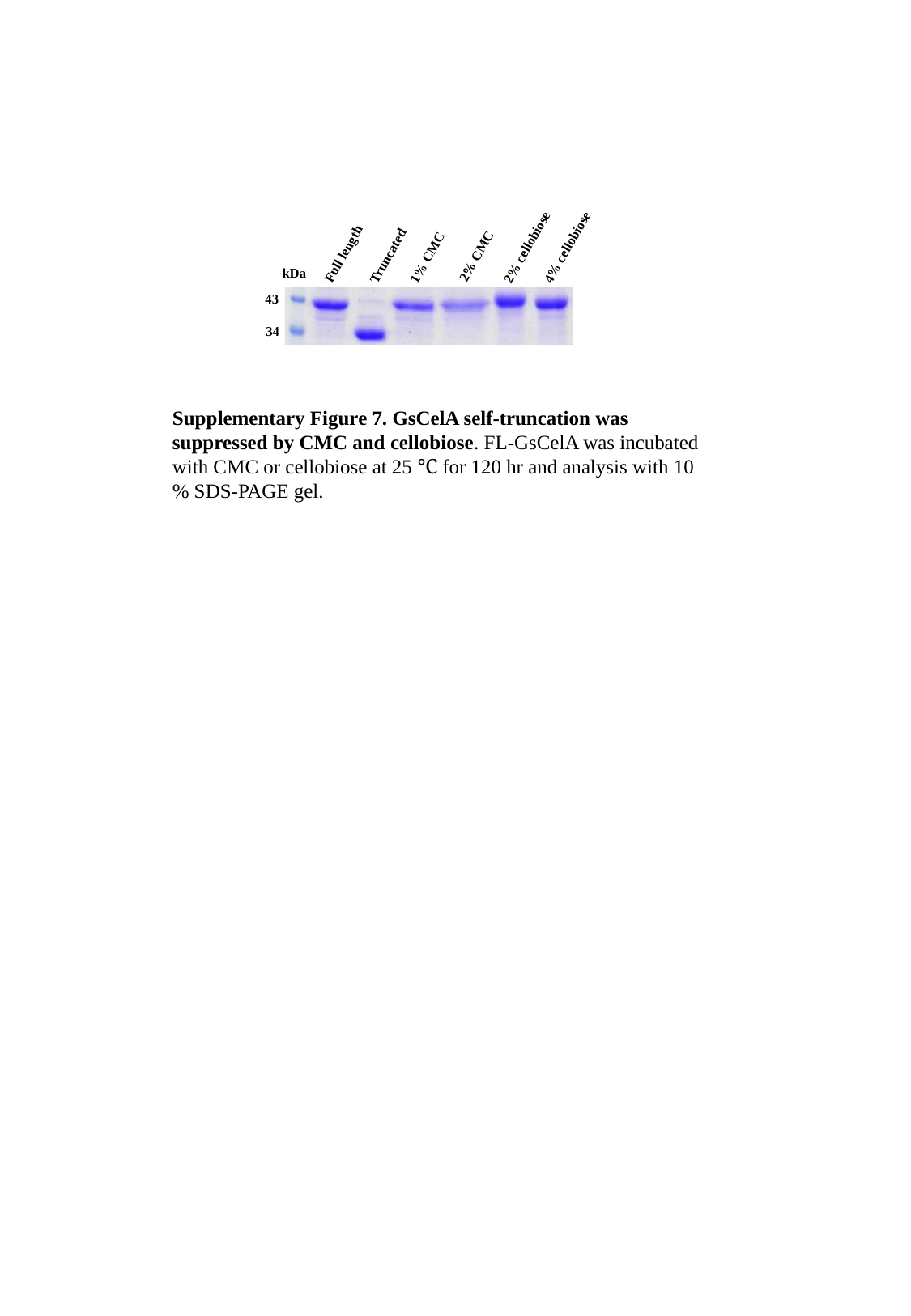

2% CMC
Full length
Truncated
1% CMC
2% cellobiose
4% cellobiose
kDa
43
34
Supplementary Figure 7. GsCelA self-truncation was suppressed by CMC and cellobiose. FL-GsCelA was incubated with CMC or cellobiose at 25 ℃ for 120 hr and analysis with 10 % SDS-PAGE gel.

## Slide 9
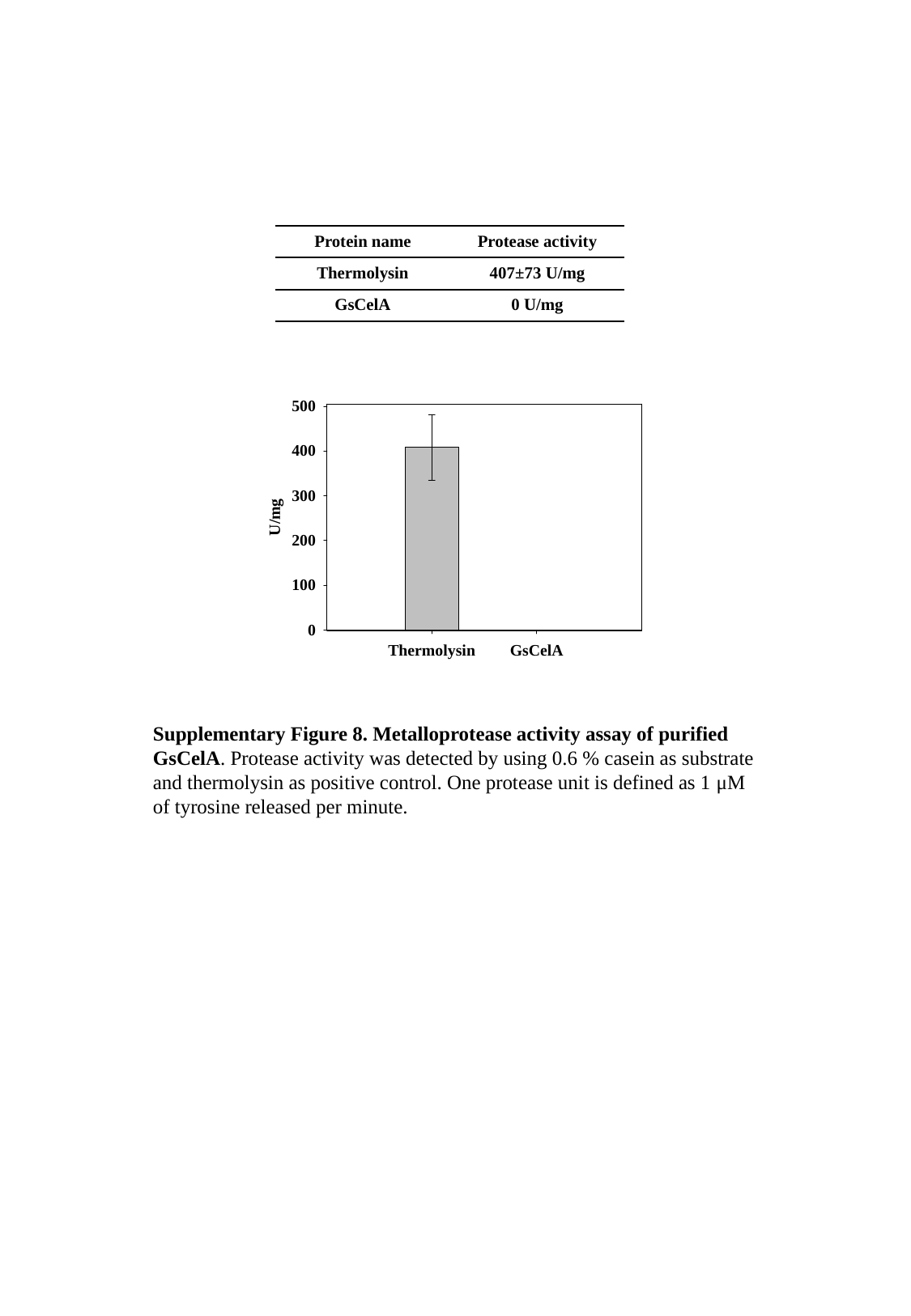

| Protein name | Protease activity |
| --- | --- |
| Thermolysin | 407±73 U/mg |
| GsCelA | 0 U/mg |
Supplementary Figure 8. Metalloprotease activity assay of purified GsCelA. Protease activity was detected by using 0.6 % casein as substrate and thermolysin as positive control. One protease unit is defined as 1 μM of tyrosine released per minute.

## Slide 10
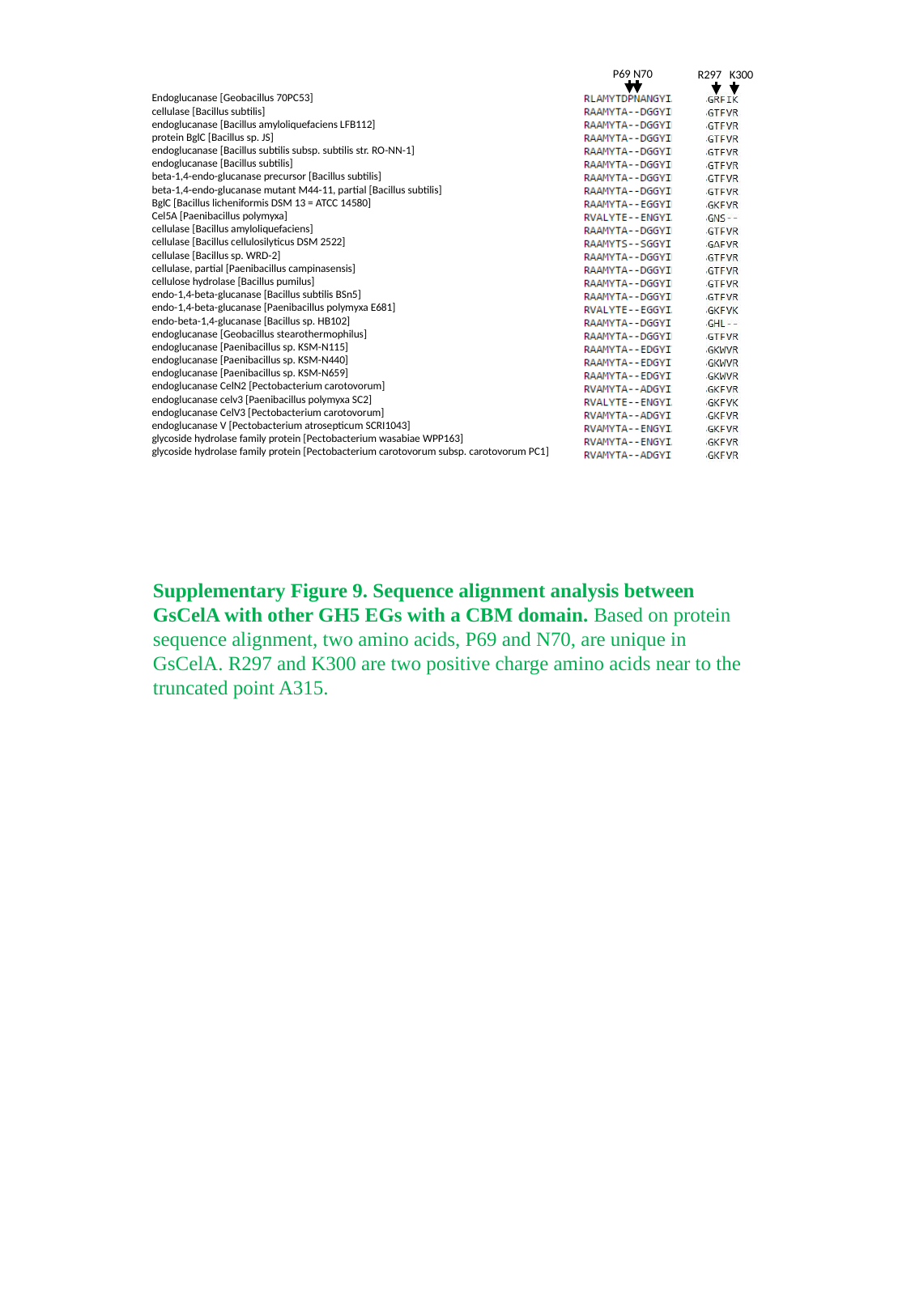

P69 N70
R297 K300
Endoglucanase [Geobacillus 70PC53]
cellulase [Bacillus subtilis]
endoglucanase [Bacillus amyloliquefaciens LFB112]
protein BglC [Bacillus sp. JS]
endoglucanase [Bacillus subtilis subsp. subtilis str. RO-NN-1]
endoglucanase [Bacillus subtilis]
beta-1,4-endo-glucanase precursor [Bacillus subtilis]
beta-1,4-endo-glucanase mutant M44-11, partial [Bacillus subtilis]
BglC [Bacillus licheniformis DSM 13 = ATCC 14580]
Cel5A [Paenibacillus polymyxa]
cellulase [Bacillus amyloliquefaciens]
cellulase [Bacillus cellulosilyticus DSM 2522]
cellulase [Bacillus sp. WRD-2]
cellulase, partial [Paenibacillus campinasensis]
cellulose hydrolase [Bacillus pumilus]
endo-1,4-beta-glucanase [Bacillus subtilis BSn5]
endo-1,4-beta-glucanase [Paenibacillus polymyxa E681]
endo-beta-1,4-glucanase [Bacillus sp. HB102]
endoglucanase [Geobacillus stearothermophilus]
endoglucanase [Paenibacillus sp. KSM-N115]
endoglucanase [Paenibacillus sp. KSM-N440]
endoglucanase [Paenibacillus sp. KSM-N659]
endoglucanase CelN2 [Pectobacterium carotovorum]
endoglucanase celv3 [Paenibacillus polymyxa SC2]
endoglucanase CelV3 [Pectobacterium carotovorum]
endoglucanase V [Pectobacterium atrosepticum SCRI1043]
glycoside hydrolase family protein [Pectobacterium wasabiae WPP163]
glycoside hydrolase family protein [Pectobacterium carotovorum subsp. carotovorum PC1]
Supplementary Figure 9. Sequence alignment analysis between GsCelA with other GH5 EGs with a CBM domain. Based on protein sequence alignment, two amino acids, P69 and N70, are unique in GsCelA. R297 and K300 are two positive charge amino acids near to the truncated point A315.

## Slide 11
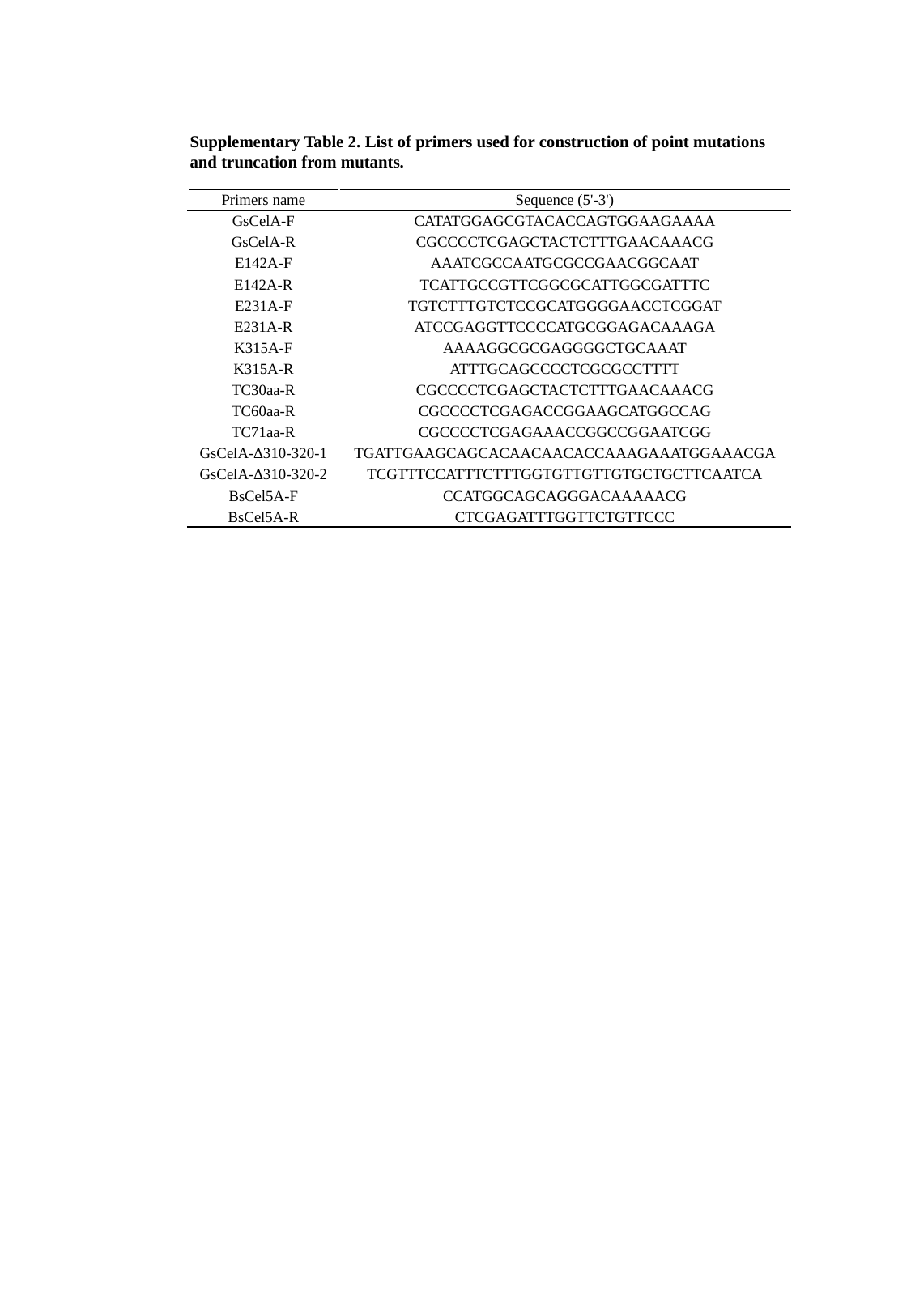

Supplementary Table 2. List of primers used for construction of point mutations and truncation from mutants.
| Primers name | Sequence (5'-3') |
| --- | --- |
| GsCelA-F | CATATGGAGCGTACACCAGTGGAAGAAAA |
| GsCelA-R | CGCCCCTCGAGCTACTCTTTGAACAAACG |
| E142A-F | AAATCGCCAATGCGCCGAACGGCAAT |
| E142A-R | TCATTGCCGTTCGGCGCATTGGCGATTTC |
| E231A-F | TGTCTTTGTCTCCGCATGGGGAACCTCGGAT |
| E231A-R | ATCCGAGGTTCCCCATGCGGAGACAAAGA |
| K315A-F | AAAAGGCGCGAGGGGCTGCAAAT |
| K315A-R | ATTTGCAGCCCCTCGCGCCTTTT |
| TC30aa-R | CGCCCCTCGAGCTACTCTTTGAACAAACG |
| TC60aa-R | CGCCCCTCGAGACCGGAAGCATGGCCAG |
| TC71aa-R | CGCCCCTCGAGAAACCGGCCGGAATCGG |
| GsCelA-Δ310-320-1 | TGATTGAAGCAGCACAACAACACCAAAGAAATGGAAACGA |
| GsCelA-Δ310-320-2 | TCGTTTCCATTTCTTTGGTGTTGTTGTGCTGCTTCAATCA |
| BsCel5A-F | CCATGGCAGCAGGGACAAAAACG |
| BsCel5A-R | CTCGAGATTTGGTTCTGTTCCC |
